# Progress and innovation of nanostructured sulfur cathodes and metal-free anodes for room-temperature Na–S batteries

**DOI:** 10.3762/bjnano.12.75

**Published:** 2021-09-09

**Authors:** Marina Tabuyo-Martínez, Bernd Wicklein, Pilar Aranda

**Affiliations:** 1 Instituto de Ciencia de Materiales de Madrid (ICMM), Consejo Superior de Investigaciones Científicas (CSIC), 28049 Madrid, Spain

**Keywords:** composites, metal-free anode, Na–S, sodium nanostructures, sodium–sulfur batteries, sulfur nanostructures

## Abstract

Rechargeable batteries are a major element in the transition to renewable energie systems, but the current lithium-ion battery technology may face limitations in the future concerning the availability of raw materials and socio-economic insecurities. Sodium–sulfur (Na–S) batteries are a promising alternative energy storage device for small- to large-scale applications driven by more favorable environmental and economic perspectives. However, scientific and technological problems are still hindering a commercial breakthrough of these batteries. This review discusses strategies to remedy some of the current drawbacks such as the polysulfide shuttle effect, catastrophic volume expansion, Na dendrite growth, and slow reaction kinetics by nanostructuring both the sulfur cathode and the Na anode. Moreover, a survey of recent patents on room temperature (RT) Na–S batteries revealed that nanostructured sulfur and sodium electrodes are still in the minority, which suggests that much investigation and innovation is needed until RT Na–S batteries can be commercialized.

## Introduction

The progress and innovation of cheaper, cleaner, safer, and more efficient electrical energy storage systems is essential for sustainable development [1–2]. Not only would it allow for a longer operation range of electronic devices such as mobile consumer electronics, electric vehicles, and stationary energy storage systems. It would also reduce fossil fuel reliance and greenhouse gas emissions if charged with “green electricity”. Therefore, improving energy storage may lead to more sustainable energy consumption [3]. In this context, rechargeable batteries play an important role owing to the fact that electrochemical energy storage is more efficient than physical energy storage [4].

Today, lithium-ion batteries (LiBs) are undoubtedly the most important mobile electrical energy storage devices. Nevertheless, they have some critical limitations such as high cost, low resource availability, as well as access and safety concerns [1,5–6]. Therefore, new promising batteries based on widely available anode and cathode materials are sought. Table 1 lists some abundant metals as anode materials with high capacity and reduction potential values that are explored in metal-ion batteries [7–9].

**Table 1:** Metals used as anode material in metal-ion batteries including their theoretic capacities, reduction potential, and abundance [7–9].

Metal	Gravimetric capacity (mAh·g^−1^)	Volumetric capacity (mAh·cm^−3^)	Reduction potential (V vs SHE)	Abundance in Earth’s crust (ppm)

Li	3862	2062	−3.04	20 (0.002%)
Na	1165	1128	−2.71	23,600 (2.36%)
K	685	591	−2.93	20,900 (2.09%)
Mg	2205	3833	−2.37	23,300 (2.33%)
Ca	2073	1337	−2.87	41,500 (4.15%)
Al	2980	8046	−1.66	82,300 (8.23%)
Zn	820	5851	−2.20	70 (0.007%)

Besides sodium as alternative anode material, also sulfur as abundant cathode material has emerged due to the high theoretical capacities of both elements (1166 mAh·g^−1^ and 1675 mAh·g^−1^, respectively), which lead to devices with high energy density [4,10]. Additionally, sulfur cathodes exhibit other advantages such as low operating voltages (1.81 V vs Na/Na^+^) and improved safety and low toxicity compared with transition-metal compounds in lithium-ion batteries [11]. Consequently, sodium–sulfur (Na–S) batteries have attracted renewed attention over the last decade due to their low cost and comparable chemistry with Li–S and LiB batteries, which would facilitate the large-scale production of Na–S batteries [3,12]. In fact, research on Na batteries is not new, starting with high-temperature (HT) Na–S batteries in the 1960s and RT sodium-ion batteries (SiBs) in the 1980s [10]. However, an appropriate anode material based on sodium was not achieved and, therefore, only lithium-ion batteries are commercially available for room-temperature applications [10].

The first commercialized Na–S battery was a high-temperature sodium–sulfur battery, which has an operational temperature in the range of 270–350 °C [13]. It was launched to the market by NGK Insulator Co. in Japan in 2002. However, these devices had important security and corrosion issues since sodium and sulfur are both liquid under the working conditions. Therefore, the applicability of HT Na–S batteries is limited to stationary deployment, and the operation temperature needs to be reduced in order to improve market penetration of Na–S batteries. As a result of scientific investigations and technological innovations, room-temperature sodium–sulfur (RT Na–S) batteries have been gaining importance since the mid-2000s [3,10,14]. A lot of effort is focused on the development of different cathode materials in order to produce commercial high-efficiency RT Na–S batteries (vide infra).

**Figure 1:**
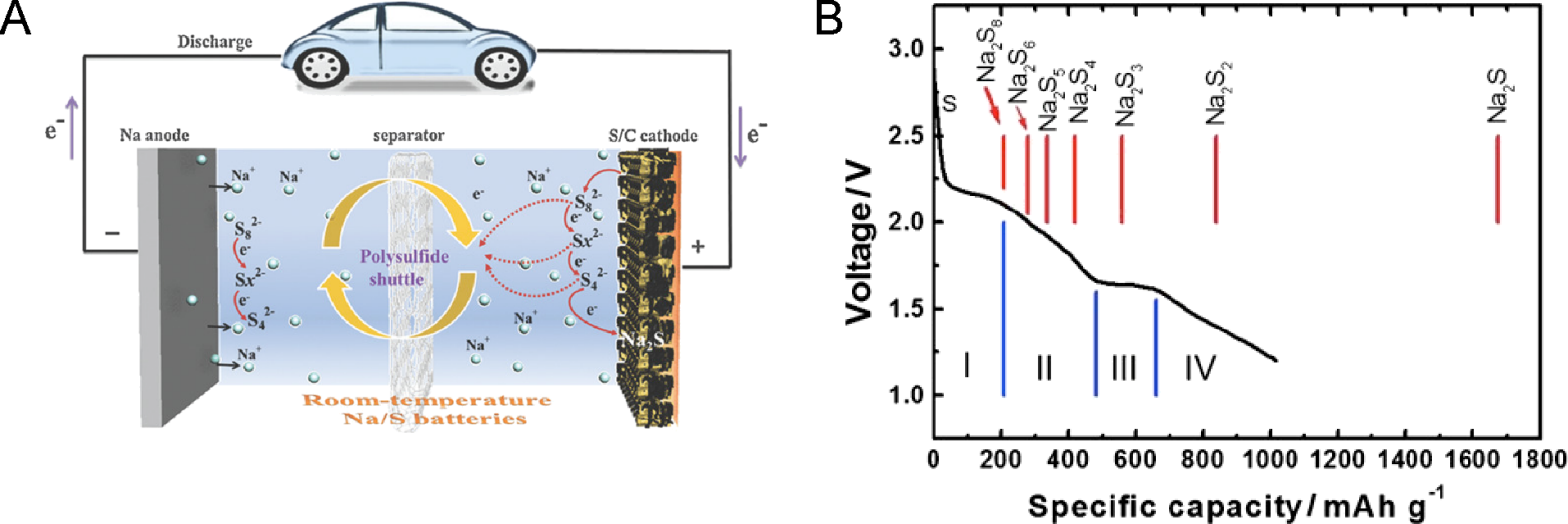
(A) Schematic representation of the electrochemical processes taking place in a RT Na–S battery. Figure 1A was reproduced with permission from [16], Y.-X. Wang et al. "Room-Temperature Sodium-Sulfur Batteries: A Comprehensive Review on Research Progress and Cell Chemistry", Adv. Energy Mater., with permission from John Wiley and Sons. Copyright © 2017 WILEY-VCH Verlag GmbH & Co. KGaA, Weinheim. This content is not subject to CC BY 4.0. (B) Theoretical discharge capacities (red lines) versus a discharge profile (black) of a representative RT Na–S cell prepared with a continuous carbon fiber interlayer. Figure 1B was reproduced with permission from [17], X. Yu et al. "Capacity Enhancement and Discharge Mechanisms of Room-Temperature Sodium–Sulfur Batteries", ChemElectroChem, with permission from John Wiley and Sons. Copyright © 2014 WILEY-VCH Verlag GmbH & Co. KGaA, Weinheim. This content is not subject to CC BY 4.0.

The electrochemical mechanism of RT Na–S batteries is based on the release of sodium cations from the anode leading to the transfer of two electrons that reduce sulfur on the cathode side (Figure 1A) [4]. The redox reactions of the battery are as follows (the discharge process corresponds to the reactions from left to right):


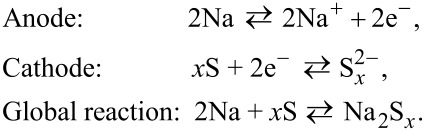


Considering the full reduction of sulfur to Na_2_S, RT Na–S batteries have a high theoretical energy density of 1276 Wh·kg^−1^ [4]. However, the reduction of sulfur to sodium sulfide is not a one-step process as it proceeds in a series of intermediate reactions [15], that is, (1) the transformation of sulfur into long-chain polysulfides: 4Na + S_8_ ⇌ 2Na_2_S*_n_* (4 < *n* ≤ 8), (2) the transformation of long-chain into short-chain polysulfides: 2Na + Na_2_S_4_ ⇌ 2Na_2_S*_m_* (2 ≤ *m* < 4), and (3) the transformation of short-chain polysulfides into sodium sulfide: 2Na + Na_2_S_2_ ⇌ 2Na_2_S. The intermediate sodium polysulfides are formed during the charge–discharge process (Figure 1B) and have important effects on the battery function, including the so-called polysulfide shuttle effect (vide infra) [4].

**Figure 2:**
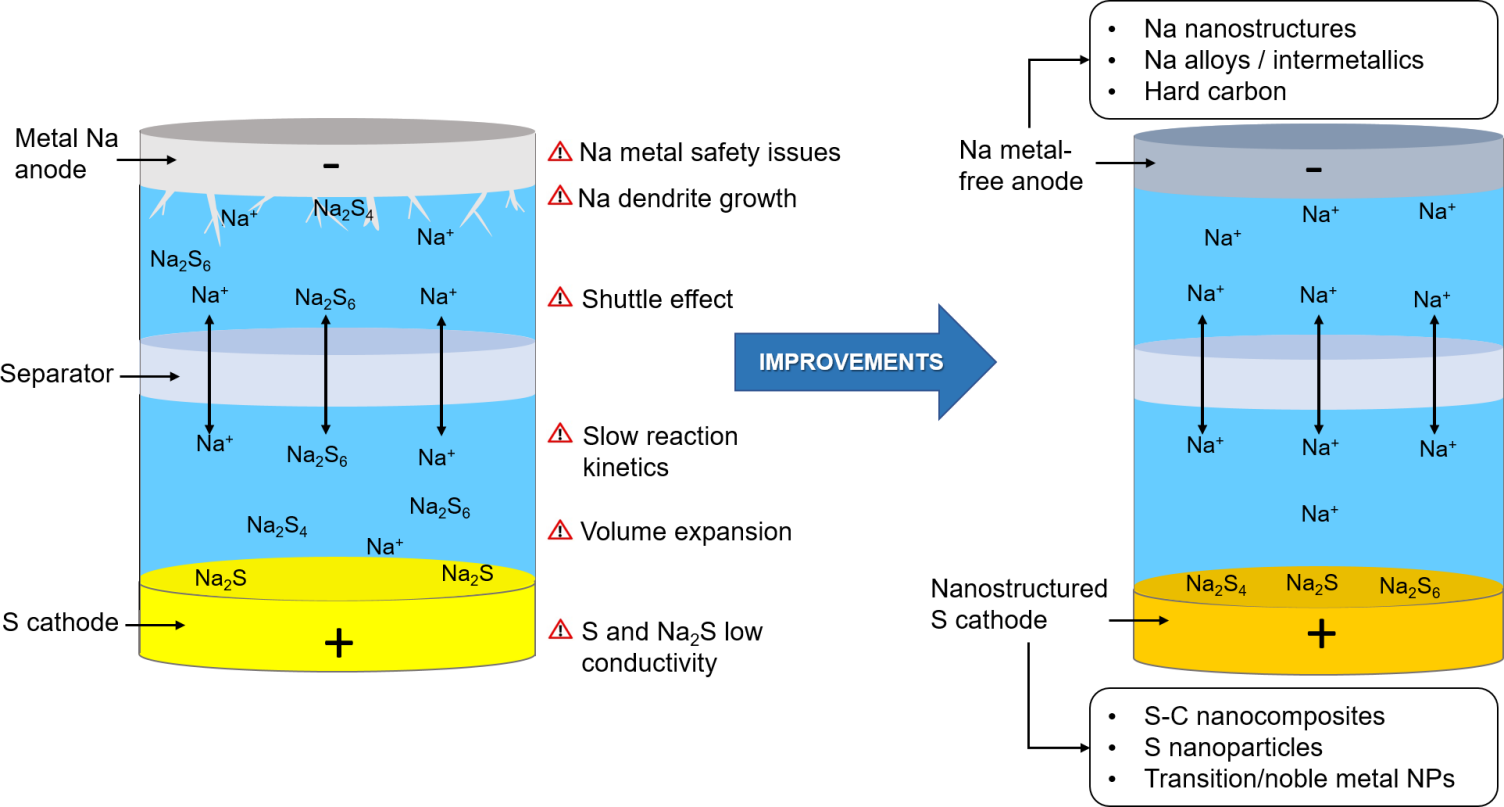
Some of the principal challenges of RT Na–S batteries and potential improvements by nanostructuring both the sulfur cathode and the Na anode as discussed below.

Technical problems of RT Na–S batteries compromise their performance and prevent their commercialization. The main drawbacks of these electrochemical devices are (1) the polysulfide shuttle effect, (2) the insulating nature of both sulfur and sodium sulfide, and (3) the large volume expansion of the cathode during the discharge process (Figure 2) [16]. Other important challenges are Na dendrite formation, capacity fading, low electrochemical utilization of sulfur, large size and mass of sodium ions, and poor understanding of the formation of discharge products [18]. In addition, replacing metal Na anodes with safer materials is another critical barrier to overcome [10,19–20]. It is essential to solve these issues to achieve high efficiency devices that can be launched onto the market.

In this review, we focus on the first three drawbacks and potential strategies, including advanced cathode materials, to overcome them. The polysulfide shuttle effect is a phenomenon caused by the migration of long-chain sodium polysulfides (Na_2_S*_n_*) to the anode, facilitated by their high solubility in carbonate- and ether-based electrolytes [21]. At the anode, Na_2_S*_n_* is further reduced to insoluble short-chain sodium polysulfides, which precipitate and passivate the electrode. These reactions are not fully reversible and lead to capacity loss of the battery [3,22] together with a low utilization of active material, low Coulombic efficiency, and poor cycle life [4,11]. Effective remedies for suppressing the shuttle effect are (1) capturing the soluble Na_2_S*_n_* within the cathode and (2) incorporating ion-selective membranes and blocking separator interlayers [14].

The insulating nature of sulfur and sodium sulfide is also problematic due to the importance of a highly conductive cathode to obtain a good performance of the RT Na–S battery. Therefore, it is necessary to design a cathode material that improves the conductivity of the system [4,11]. Common approaches are based on the incorporation of conductive carbon nanomaterials [23].

The volume expansion of sulfur during the discharge process is caused by the formation of the discharge product Na_2_S. This expansion is rather large (up to 170%) and may damage the cathode and lead to capacity loss [4]. Nanoscaling the cathode compounds and the concomitant introduction of porosity is a widely investigated strategy to mitigate the emerging mechanical tension and to prevent electrode failure [18,24].

Unlike sodium-ion batteries, the anode of most RT Na–S batteries simply consists of a Na metal foil. This has severe security and technological implications as metallic Na is highly reactive, prone to dendrite growth and subsequent mechanical failure, and reduced cycle performance [10]. These inconveniences are detrimental to the expansion of these batteries into wider consumer markets, for which hard carbon, Si, Sn and Sb alloys, as well as phosphorous compounds are currently investigated [25–27].

This review focuses on the most recent designs of cathode materials for RT Na–S batteries, which attempt to overcome the drawbacks of sulfur-based cathodes. The strategies to solve the polysulfide shuttle effect, conductivity drop, and structural damage caused by sulfur volume expansion are discussed. Moreover, concepts for Na metal-free anodes in Na–S batteries are reviewed and analyzed. Other strategies including electrolyte engineering, cell design, interlayers, or solid electrolyte interphases can be found elsewhere in excellent reviews [10,14,28]. Here, additionally, some patents are reviewed to examine the approaches that are followed to commercialize Na–S batteries. Finally, an outlook is provided on how far this technology has currently developed and where future research could be directed at.

## Review

### Conventional sulfur–carbon cathode materials

Sulfur–carbon composites are the most widely studied cathode materials because carbon increases the cathode conductivity and also improves the reactivity of sulfur [4]. Since carbon structures are highly diverse, a huge variety of cathode materials have been designed and tested in RT Na–S batteries. Here, the sulfur–carbon composites are classified in two main categories: (1) sulfur–porous carbon composites and (2) covalently bound sulfur–carbon composites.

#### Sulfur–porous carbon composites

**Figure 3:**
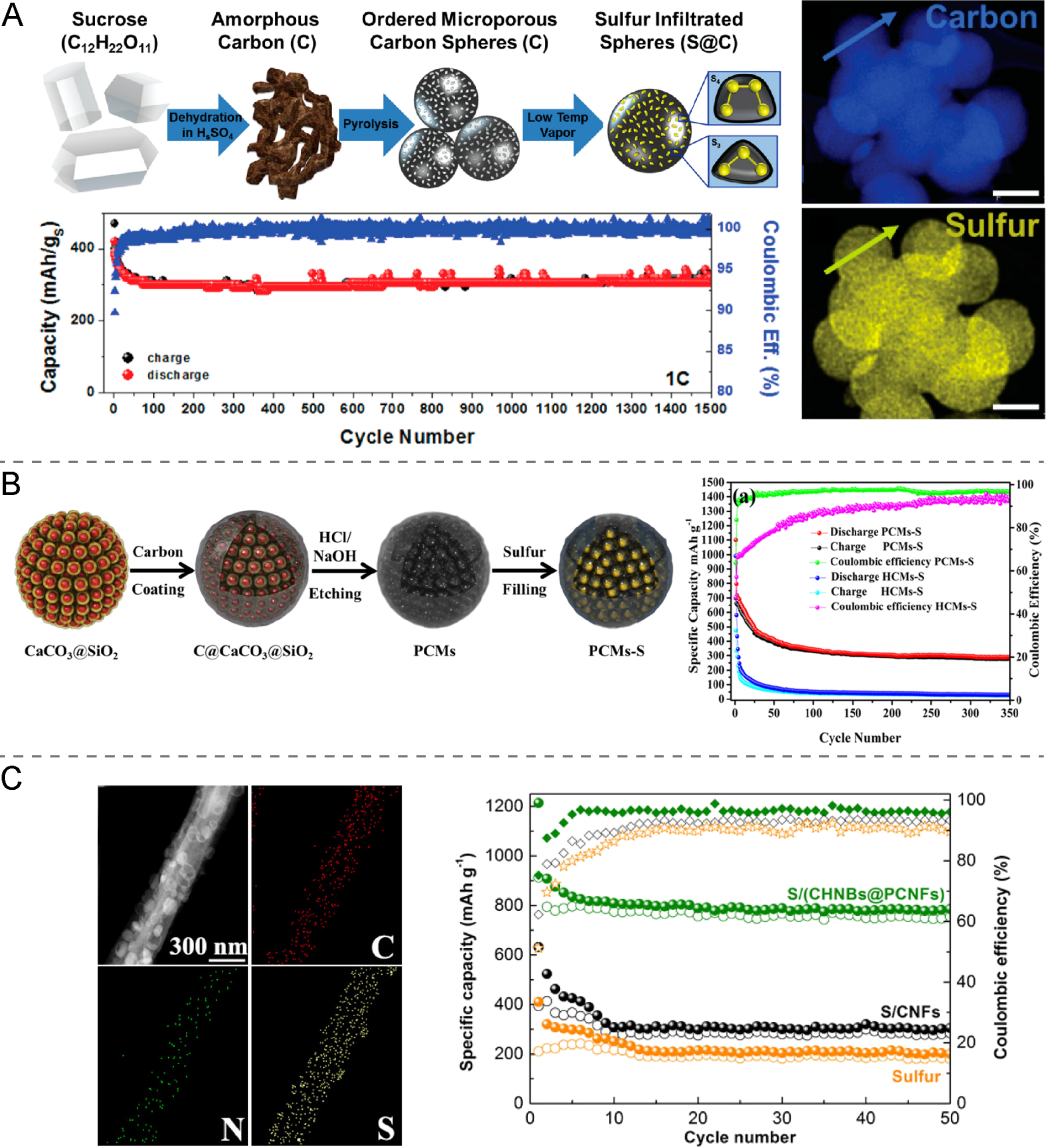
(A) Schematic illustration of the processing steps of using sucrose to produce microporous, sulfur-infiltrated carbon spheres (S@C). The STEM EDS maps of S@C show carbon (blue) and sulfur (yellow). The capacity and Coulombic efficiency plotted over 1500 cycles at 1C. Figure 3A was reprinted with permission from [29]. Copyright 2017, The American Chemical Society. This content is not subject to CC BY 4.0. (B) Schematic illustration of the fabrication process of porous carbon microspheres filled with sulfur (PCMs–S) and the respective cycling properties of double- and single-shell carbon microspheres (PCMs-S vs HCMs-S) at 100 mA·g^−1^. Figure 3B was reprinted with permission from [24]. Copyright 2018, The American Chemical Society. This content is not subject to CC BY 4.0. (C) Scanning STEM image and the corresponding elemental mapping of S containing carbon hollow nanobubbles on porous carbon nanofibers and the cycling performance at a current density of 0.1C. Figure 3C was reprinted from [32], Energy Storage Materials, vol. 14, by G. Xia, L. Zhang, X. Chen, Y. Huang, D. Sun, F. Fang, Z. Guo, X. Yu, "Carbon hollow nanobubbles on porous carbon nanofibers: An ideal host for high-performance sodium-sulfur batteries and hydrogen storage", pages no. 314-323. Copyright (2018), with permission from Elsevier. This content is not subject to CC BY 4.0.

Hollow and porous carbon structures may not only increase cathode conductivity, but can also allow for physical confinement of long-chain sodium polysulfides and reduce the structural damage caused by sulfur volume expansion [4,11]. This makes sulfur–porous carbon composites remarkably promising cathode materials for RT Na–S batteries. Among the different studied materials are, for instance, microporous (Figure 3) and ultramicroporous carbon materials, which have shown a considerable ability to confine sulfur and sodium polysulfides [29–30]. This confinement significantly improves the cycling stability since the shuttle effect is minimized. For instance, a capacity of 300 mAh·g^−1^ after 1500 cycles at 1C and a Coulombic efficiency of 98% can be achieved (Figure 3A) [29].

Furthermore, hierarchical porous carbon structures have also shown promising performance as sulfur host material [24,31]. As an example, Zhang et al*.* [24] designed a sulfur host based on porous double-shell microspheres, which consist of hollow carbon nanobeads inside a microsized carbon shell. In this structure, sulfur is infused in the nanobeads inside the microspheres and neither sulfur nor sodium polysulfide species are directly exposed to the electrolyte. Additionally, the hollow structure provides space to accommodate the volume expansion of sulfur during the discharge processes. This cathode composite limits the shuttle effect, increases utilization and activity of sulfur, and prevents cathode damage due to the volume change of sulfur. Therefore, an improvement in charge capacity (300 vs 50 mAh·g^−1^) and cycling stability is achieved when comparing the double- with single-shell carbon microspheres as shown in Figure 3B.

In addition, modified porous carbon structures with nitrogen or oxygen doping have proven to enhance the immobilization of sodium polysulfides leading to an advancement in battery performance [31–32]. Adsorption and trapping of polysulfides are achieved through strong interactions between the sodium atoms in sodium polysulfides and the nitrogen and oxygen atoms. The shuttle effect is therefore diminished, which results in an improvement in cycling stability. In this way, Qiang et al. [31] reported a decay in discharge capacity of only 3% after 8000 cycles at a high current density of 4.6 A·g^−1^. This improvement has also been clearly shown in the electrochemical performance of the RT Na–S battery reported by Xia et al*.* [32] who used nitrogen-doped hollow carbon nanobubbles supported on porous carbon nanofibers as sulfur hosts. The nitrogen content of this carbonaceous structure is shown by a mapping image in Figure 3C. When studying the electrochemical properties of this material, improved results are obtained with a cycle life of up to 400 cycles and a small capacity decay rate of 0.044% per cycle.

Moreover, Chen et al. [33] reported the potential use of metal-organic frameworks as cathode skeleton. Herein, a sulfur host based on a nanoporous nitrogen-doped carbon matrix was obtained through carbonization of a zeolitic imidazolate framework (ZIF-8). The cathode exhibits good performance with a reversible specific capacity of 500 mAh·g^−1^ after 250 cycles at 0.2C. The excellent electrochemical behavior is based on the efficient polysulfide entrapment as result of the nanoporosity of the carbon matrix and the high nitrogen-doping content (ca. 18 atom %).

Among all sulfur–carbon composite cathodes, flexible carbon-based skeletons are one of the most promising cathode materials given their ability to accommodate the fast volume changes of sulfur during the discharge process. Ma et al*.* [34] reported a conductive and flexible graphene aerogel cathode that effectively tolerated the volume changes under stabilization of the structure and led to an outstanding performance, showing an initial discharge capacity of 572.8 mAh·g^−1^ at 5C and an extremely low average capacity fading.

#### Covalently bound sulfur–carbon cathodes

**Figure 4:**
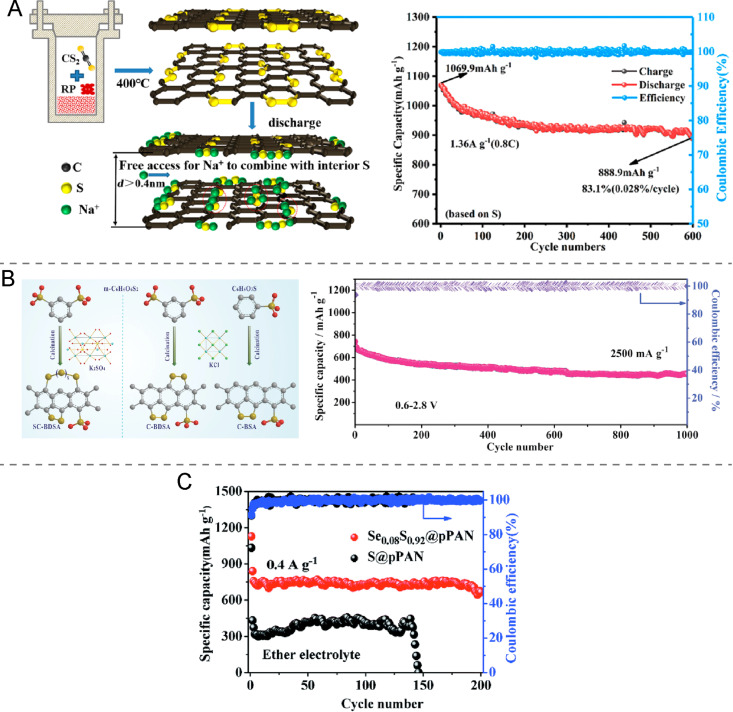
(A) Schematic illustration of preparation and structure of the covalent sulfur–carbon composite synthesized with CS_2_ and red phosphorus and the corresponding long cycle performance at 0.8C. Figure 4A was reprinted with permission from [36]. Copyright 2020, The American Chemical Society. This content is not subject to CC BY 4.0. (B) Schematic representation of the synthesis processes of C‐BSA, C‐BDSA, and SC‐BDSA and the cyclic performance and Coulombic efficiency of the activated SC‐BDSA electrode for Na–S battery system at 2500 mA·g^−1^ for 1000 cycles. Figure 4B was reprinted with permission from [37], T. Wu et al. "Controllable Chain-Length for Covalent Sulfur–Carbon Materials Enabling Stable and High-Capacity Sodium Storage", Adv. Energy Mater., with permission from John Wiley and Sons Copyright © 2019 WILEY-VCH Verlag GmbH & Co. KGaA, Weinheim. This content is not subject to CC BY 4.0. (C) Cycling performance of Se_0.08_S_0.92_@pPAN and S@pPAN in an ether electrolyte. Figure 4C was republished with permission of The Royal Society of Chemistry, from [38] ("Effect of eutectic accelerator in selenium-doped sulfurized polyacrylonitrile for high performance room temperature sodium–sulfur batteries" by L. Wang et. al., J. Mater. Chem. A, vol. 7, © 2019); permission conveyed through Copyright Clearance Center, Inc. This content is not subject to CC BY 4.0”.

Cathodes with sulfur covalently bound to carbon are promising materials because the strong sulfur–carbon bond prevents polysulfides from dissolving and migrating, thus mitigating the shuttle effect. Therefore, an enhancement of the battery cycling stability can be achieved. Many different materials might be used as cathode skeleton. As an example, Huo et al. [35] reported the use of a two-dimensional layered material Ti_3_C_2_T*_x_* (where T*_x_* are surface functional groups such as F_2_ and (OH)_2_) into which sulfur was inserted by a simple melt/sublimation process forming C–S bonds within the MXene. This cathode shows reasonable cycle stability (150 mAh·g^−1^ after 300 cycles at 100 mA·g^−1^), however the sulfur loading process needs to be optimized to obtain a RT Na–S battery with higher capacity values. Yan et al. [36] reported a covalent sulfur–carbon composite prepared from CS_2_ (Figure 4A) that delivers high reversible capacities of 889 mAh·g^−1^ after 600 cycles at 0.8C and of 811 mAh·g^−1^ after 950 cycles at 1.6C. Additionally, Wu et al. [37] reported a cathode material based on a covalent sulfur–carbon complex with a high concentration of covalently bonded sulfur (40.1%), which displays a specific capacity of 696 mAh·g^−1^ at 2.5 A·g^−1^. Unlike most of the reported cathodes, where sulfur is infused in its elemental form (S_8_), in this design the sulfur source are benzenedisulfonic acid (BDSA, –SO_3_H) and sulfate (SO_4_^2−^), which are shown in Figure 4B. Moreover, the carbon structure in the cathode has mesopores and, therefore, can confine a certain amount of sulfur and polysulfides. In addition to the confinement, the generated polysulfides can also be anchored by the partially oxidated sulfur–carbon units (R-SO) and form insoluble surface-bound intermediates. Consequently, the shuttle effect is minimized resulting in excellent cycle stability for 1000 cycles with 0.035% capacity decay per cycle.

The research based on covalently bonded sulfur–carbon cathodes has been mostly focused on electrodes with a polyacrylonitrile (PAN) skeleton [38–41]. For instance, Hwang et al. [39] reported a cathode material based on one-dimensional sulfurized PAN nanofibers, and Kim et al. [40] reported the design of a flexible cathode that consists of a sulfurized PAN nanofiber web. The sulfurized PAN web was prepared by pyrolysis of PAN nanofibers and elemental sulfur at 450 °C for 6 h in an inert gas atmosphere. Both electrodes exhibit an excellent cycling performance because the shuttle effect and the low conductivity of elemental sulfur are avoided [39–40]. However, its applicability is hindered due to the low capacity values achieved. The former cathode is reported to display a capacity of 153 mAh·g^−1^ after 500 cycles at 1C [39], while the latter has 257 mAh·g^−1^ after 200 cycles at 1C [40]. This problem is a consequence of the low sulfur content of the polymer, which is usually less than 50 wt % and the limited redox reactivity of sulfur [38,41]. In order to overcome the consequences of the limited reactivity, a small amount of selenium can be added to the cathode and uniformly distributed through selenium–sulfur bonds. A significant improvement in redox reaction kinetics is achieved since selenium has a higher conductivity than sulfur and acts as an accelerator [38]. Therefore, the resulting cathode exhibits an improved cycling performance as shown in Figure 4C. A high specific capacity of 770 mAh·g^−1^ after 200 cycles at 0.4 A·g^−1^ with a small capacity decay per cycle of 0.045% was obtained [38].

Additionally, Li et al. [41] reported a pyrolyzed PAN/SeS_2_ composite that results in a cathode with excellent reaction kinetics, high capacity, and extremely stable cycle life. The multichannel framework of the composite provides more surface area of PAN to react with SeS_2_, leading to an enhancement of the sulfur load on the cathode to about 63 wt % of SeS_2_. As a result, the cathode exhibits a reversible capacity of 800 mAh·g^−1^ after 400 cycles at 1 A·g^−1^.

### Sodium polysulfide composites as cathodes

**Figure 5:**
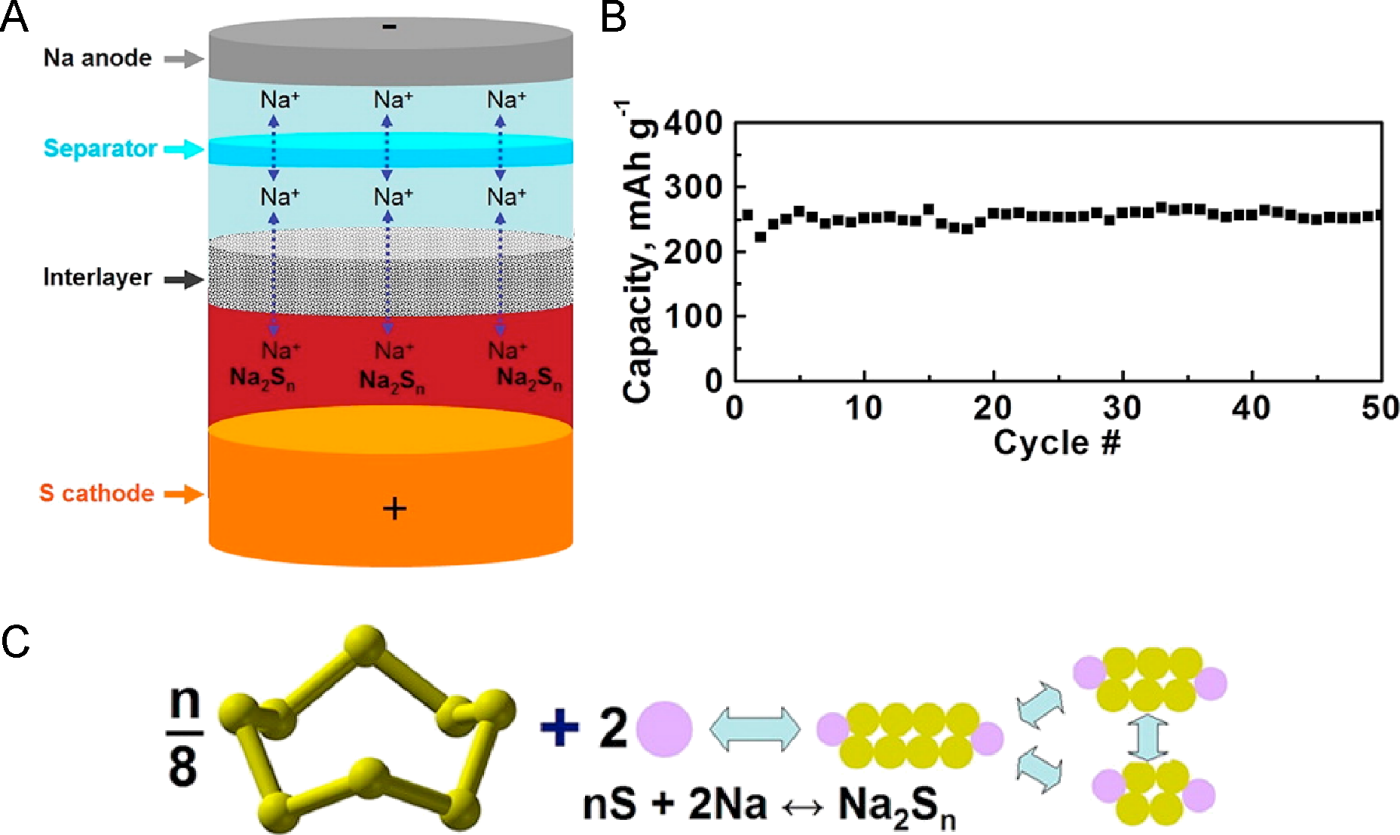
(A) Schematic representation of a RT Na–S battery with a liquid-phase catholyte containing polysulfides and an interlayer separating the anode from the cathode. (B) Discharge capacity of the sulfur/long-chain sodium polysulfide battery over 50 cycles. (C) Illustration of the reaction mechanism of S_8_ and Na to long-chain sodium polysulfide. Figure 4A–C was reprinted with permission from [42]. Copyright 2014, The American Chemical Society. This content is not subject to CC BY 4.0.

Long-chain sodium polysulfide composites emerged as an alternative to cathode composites using elemental sulfur as active material. This is because both Na_2_S_8_ and the reduction products, Na_2_S*_n_* (4 < *n* < 8), show higher electrical conductivity than elemental sulfur and sodium sulfide Na_2_S and faster reaction kinetics with Na^+^ [42]. Therefore, by employing the S/Na_2_S*_n_* redox couple as cathode, the electrode conductivity is enhanced, which improves the discharge efficiency and the sulfur utilization rate. In this battery design, the cathode reactions do not proceed to sodium sulfide by properly adjusting the discharge cut-off voltage, avoiding low conductivity and irreversibility problems. However, a significant disadvantage of these composites is the fact that sodium polysulfides are incorporated as a liquid phase (so-called catholyte) and thus, it is necessary to confine them at the cathode. Otherwise, they would migrate to the anode and compromise the battery performance via the shuttle effect. Figure 5 shows an illustration of the resulting RT Na–S battery and the cycle performance. Another drawback is the lower theoretical cell capacity by not discharging until Na_2_S is formed.

One approach to physically confine the catholyte is using membranes. Cengiz et al*.* [43] reported a RT Na–S battery with a Na_2_S_5_ catholyte confined by a Al_2_O_3_–Nafion membrane. Using this barrier, the capacity retention could be improved to 250 mAh·g^−1^ after 100 cycles. In spite of the improvement, the capacity value is still low because of the low Na^+^ diffusivity through the membrane and the insulating nature of Al_2_O_3_. Another approach to stable catholytes is combining the active material with free-standing, cloth-type carbonaceous materials. For instance, Yu et al*.* [22] reported the development of a cathode based on a sodium polysulfide catholyte soaked into a multiwall carbon nanotube (MWCNT) fabric. The resulting fabric/Na_2_S_6_ cathode exhibited higher capacity retention during cycling and higher active material utilization when compared with traditional sulfur–carbon composites. Additionally, using sodium polysulfides facilitates the dispersion and homogeneous distribution of sulfur into the nanostructured MWCNT matrix, which acts as a high-surface current collector. As a result, the sodium polysulfide/MWCNT fabric cathode delivers a discharge capacity of 400 mAh·g^−1^ after 30 cycles.

### Transition metal nanoparticles as polysulfide sequestrants and electrocatalysts

As discussed above, hollow and porous carbonaceous structures, and in particular nitrogen or oxygen-doped carbon-based materials, are able to physically confine sodium polysulfides, minimizing the shuttle effect. However, the interaction between the carbon structure and sodium polysulfides is generally weak since the former is a nonpolar material while the latter is a polar compound [12]. Therefore, the shuttle effect often cannot be avoided. In order to solve this limitation, transition metal nanoparticles (NPs) or compounds are incorporated in the cathode. The metallic species is bonded to the carbon skeleton creating a dipole, which leads to dipole–dipole interactions with the polysulfides. Thus, the sulfur host has stronger affinity to polysulfides, which get highly immobilized in the cathode.

Thus, Zheng et al. [44] reported on a cathode in which the shuttle effect is completely prevented. The sulfur host is based on copper nanoparticles deposited on high surface area mesoporous carbon (HSMC). The resulting electrode exhibited a Coulombic efficiency of 100% and a capacity of 610 mAh·g^−1^ after 110 cycles at 0.03C. This improved cycling stability was attributed to the conductivity enhancement imparted by copper, the sulfur immobilization due to strong copper–polysulfide interactions, and the free space for volume expansion that is provided by the HSMC matrix.

In addition to sequestering polysulfides, transition metals may also act as electrocatalysts in the reduction reaction of long-chain sodium polysulfides into short-chain sodium polysulfides or sodium sulfide. Consequently, the electrocatalysts accelerate the reaction kinetics, improving the electrochemical performance of Na–S batteries. Different compounds were shown to have this property such as cobalt nanoparticles [34,45–46], iron nanoclusters [47] and iron disulfide [48], gold nanodots [49], nickel sulfide nanocrystals [50], molybdenum trioxide [21], manganese dioxide [51], and vanadium carbide nanoparticles [12].

**Figure 6:**
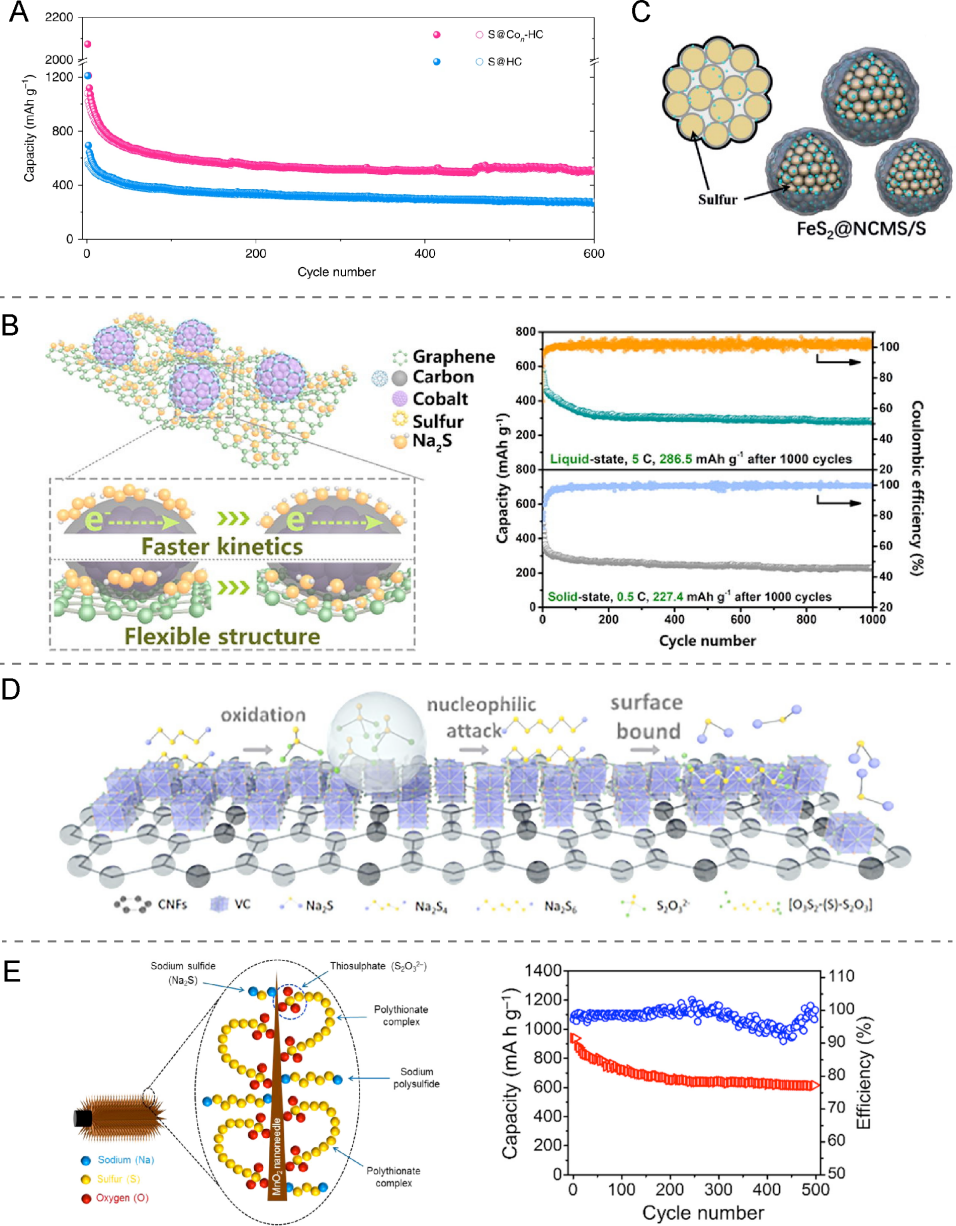
(A) Cycling performance of a sulfur–hollow carbon nanospheres cathode composite with and without cobalt nanoparticles. Figure 6A was reproduced from [45] (© 2018 B. Zhang et al., published by Springer Nature, distributed under the terms of the Creative Commons Attribution 4.0 International License, https://creativecommons.org/licenses/by/4.0). (B) Schematic illustration of the S@Co/C/rGO composite electrode and its cycling performance in solid- and liquid-state RT/Na–S batteries. Figure 6B was reprinted from [34], Chemical Engineering Journal, vol. 388, by Q. Ma, G. Du, B. Guo, W. Tang, Y. Li, M. Xu, C. Li, "Carbon-wrapped cobalt nanoparticles on graphene aerogel for solid-state room-temperature sodium-sulfur batteries", article no. 124210. Copyright (2020), with permission from Elsevier. This content is not subject to CC BY 4.0. (C) Schematic illustration of the hierarchical FeS_2_@NCMS/S composite. Figure 6C was reproduced with permission from [48], Z. Yan et al. "A High-Kinetics Sulfur Cathode with a Highly Efficient Mechanism for Superior Room-Temperature Na–S Batteries", Adv. Mater., with permission from John Wiley and Sons Copyright © 2020 WILEY-VCH Verlag GmbH & Co. KGaA, Weinheim. This content is not subject to CC BY 4.0. (D) Schematic diagram on the catalytic mechanism of the VC-CNFs composite with Na_2_S_6_. Figure 6D was reprinted from [12], Chemical Engineering Journal, vol. 395, by W. Tang, W. Zhong, Y. Wu, Y. Qi, B. Guo, D. Liu, S-J. Bao, M. Xu, "Vanadium carbide nanoparticles incorporation in carbon nanofibers for room-temperature sodium sulfur batteries: Confining, trapping, and catalyzing", article no. 124978. Copyright (2020), with permission from Elsevier. This content is not subject to CC BY 4.0. (E) Schematic representation of the interactions of MnO_2_ with different sodium polysulfides and polythionate complexes as well as the cycling performance of CC@MnO_2_@Na_2_S_6_ at 0.2 A·g^−1^. Figure 6E was reprinted from [51], Energy Storage Materials, vol. 20, by A. Kumar, A. Ghosh, A. Roy, M. R. Panda, M. Forsyth, D. R. MacFarlane, S. Mitra, "High-energy density room temperature sodium-sulfur battery enabled by sodium polysulfide catholyte and carbon cloth current collector decorated with MnO_2_ nanoarrays", pages no. 196-202. Copyright (2019), with permission from Elsevier. This content is not subject to CC BY 4.0.

For instance, the electrocatalytic performance of cobalt nanoparticles (CoNPs) was reported by Zhang et al. [45] who studied a cathode comprising hollow carbon nanospheres. The cathode displayed a capacity of 271 mAh·g^−1^ after 600 cycles at 0.1 A·g^−1^ before incorporating CoNPs and a value of 508 mAh·g^−1^ after the addition (Figure 6A), suggesting the electrocatalytic role of CoNPs. Likewise, Du et al. [46] described a cathode with a skeleton based on nitrogen-doped porous carbon nanofibers and CoNPs that exhibited a capacity of 906 mAh·g^−1^ at 0.1C and a long cycling life. Another carbon–Co structure was reported by Ma et al. [34] who developed a cathode made of a flexible graphene aerogel matrix that shows an extremely low capacity fading of 0.01% per cycle from 200 to 1000 cycles (Figure 6B). This performance was attributed to the enhanced reaction kinetics caused by the incorporated CoNPs and the accommodation of volume changes enabled by the flexible aerogel.

Also, transition metal nanoclusters that are smaller than nanoparticles were shown to enhance sulfur reactivity and avoid the shuttle effect. Zhang et al. [47] studied iron, copper, and nickel nanoclusters (ca 1.2 nm) loaded onto hollow carbon nanospheres. On the one hand, the chemical coupling between nanocluster and sulfur assists in sulfur immobilization and enhances conductivity and reactivity. On the other hand, the electrocatalytic performance of the nanoclusters reduces long-chain polysulfides to short-chain polysulfides avoiding the shuttle effect. Among all of them, the iron nanoclusters displayed the most outstanding reversible capacity of initially 1023 mAh·g^−1^ and 394 mAh·g^−1^ after 1000 cycles at 0.1 A·g^−1^.

Yan et al. [48] also reported a promising cathode with iron in the form of iron disulfide nanoparticles as shown in Figure 6C. In order to explain the electrocatalytic behavior of FeS_2_, a two-step mechanism is proposed. Firstly, polysulfides are adsorbed on the surface of FeS_2_ NPs by strong chemical bonds and undergo a sodiation process to form Na_2_S_2_. Then, the Na_2_S_2_ intermediate is converted to Na_2_S. Another remarkable electrocatalyst are gold nanodots, as reported by Wang et al. [49]. The in situ synchrotron XRD results show that gold can effectively catalyze the transformation of Na_2_S_4_ into Na_2_S in the discharge process and of Na_2_S_4_ into elemental sulfur in the charging process. Therefore, a complete conversion of polysulfides is achieved in both charge and discharge processes leading to an extraordinary high cycling stability (430 mAh·g^−1^ after 1000 cycles at 2 A·g^−1^ and 369 mAh·g^−1^ after 2000 cycles at 10 A·g^−1^) [49].

Metallic and metal oxide compounds have also attracted much interest due to their electronic conductivity and their high polarity, which leads to strong chemical interaction with polysulfides. For instance, Yan et al*.* [50] reported an electrode with an excellent performance that is based on nickel disulfide nanocrystals implanted in nitrogen-doped porous carbon nanotubes. It exhibits high reversible capacity of 650 mAh·g^−1^ after 200 cycles at 0.1 A·g^−1^ and excellent cycling stability for 3500 cycles. Additionally, Kumar et al. [51] described a cathode, based on manganese dioxide in a carbon cloth as sulfur host and Na_2_S_6_ catholyte as active material, that showed a reversible capacity of 610 mAh·g^−1^ after 500 cycles at 0.2 A·g^−1^.

Likewise, a cathode where sulfur is used as the core layer and MoO_3_ is used as the catalytic shell layer was designed by Vijaya Kumar Saroja and co-workers [21]. Molybdenum trioxide prevents polysulfides from migrating due to its electrocatalytic and chemical sequestrant character. Therefore, a cycling stability of 2000 cycles was achieved. In the same way, a cathode with high capacity retention of 96.2% after 2000 cycles was presented by Tang and co-workers [12]. The electrode described is a three-dimensional self-supported structure with vanadium carbide nanoparticles embedded in carbon nanofibers.

Since these compounds are promising regarding efficient batteries, increased understanding of the mechanism of the electrocatalytic processes is essential to further develop cathode materials containing metallic species. As noted above, Tang et al. [12] reported that vanadium carbide nanoparticles capture long-chain polysulfides and convert them into short-chain polysulfides through the catalytic mechanism schematically shown in Figure 6D. First, the vanadium carbide–carbon nanofibers (VC-CNFs) composite acts as electrocatalyst for oxidizing polysulfides to thiosulfate. Second, the thiosulfate serves as mediator to immobilize long-chain sodium polysulfides and transforming them to short-chain sodium polysulfides or sodium disulfide. Then, polysulfides are converted to polythionate [O_3_S_2_–(S)*_x_*_−2_–S_2_O_3_] complexes bound to the electrode surface, which inhibits the shuttle effect [12]. A similar electrochemical mechanism has been reported by Kumar et al. [51] based on XPS analyses. The results show that the interaction between MnO_2_ and long-chain polysulfides is not only electrostatic but also involves surface redox reactions. The polysulfides are oxidized to thiosulfate by MnO_2_ while Mn(IV) is reduced to Mn(III) and Mn(II). Afterwards, the formed thiosulfate interacts with long-chain polysulfides and converts them into short-chain polysulfides. The interactions between the metallic compound and the sulfur species are schematically shown in Figure 6E.

### Sulfur nanoparticles and nanostructures

As described above, sulfur has become a very promising cathode material in RT alkali batteries due to its high theoretical capacity. Yet, sulfur in its bulk form presents severe obstacles to a more widespread use, especially in commercial alkali–sulfur batteries. The major electrochemical challenges of bulk sulfur are low electrical conductivity, large volume expansion on discharge (S → Na_2_S), slow reaction kinetics with Na, formation and loss of polysulfides due to the shuttle effect, low electrochemical utilization of sulfur, and low specific surface area [16,52]. Many of these drawbacks can be overcome by reducing the size of the active sulfur phase to the nanoparticle range. The accompanied increase in surface area generally accelerates interfacial reaction kinetics, NPs are more easily confined in conducting matrices, and the volume expansion of individual NPs is better distributed over the entire cathode improving its mechanical integrity [14].

**Figure 7:**
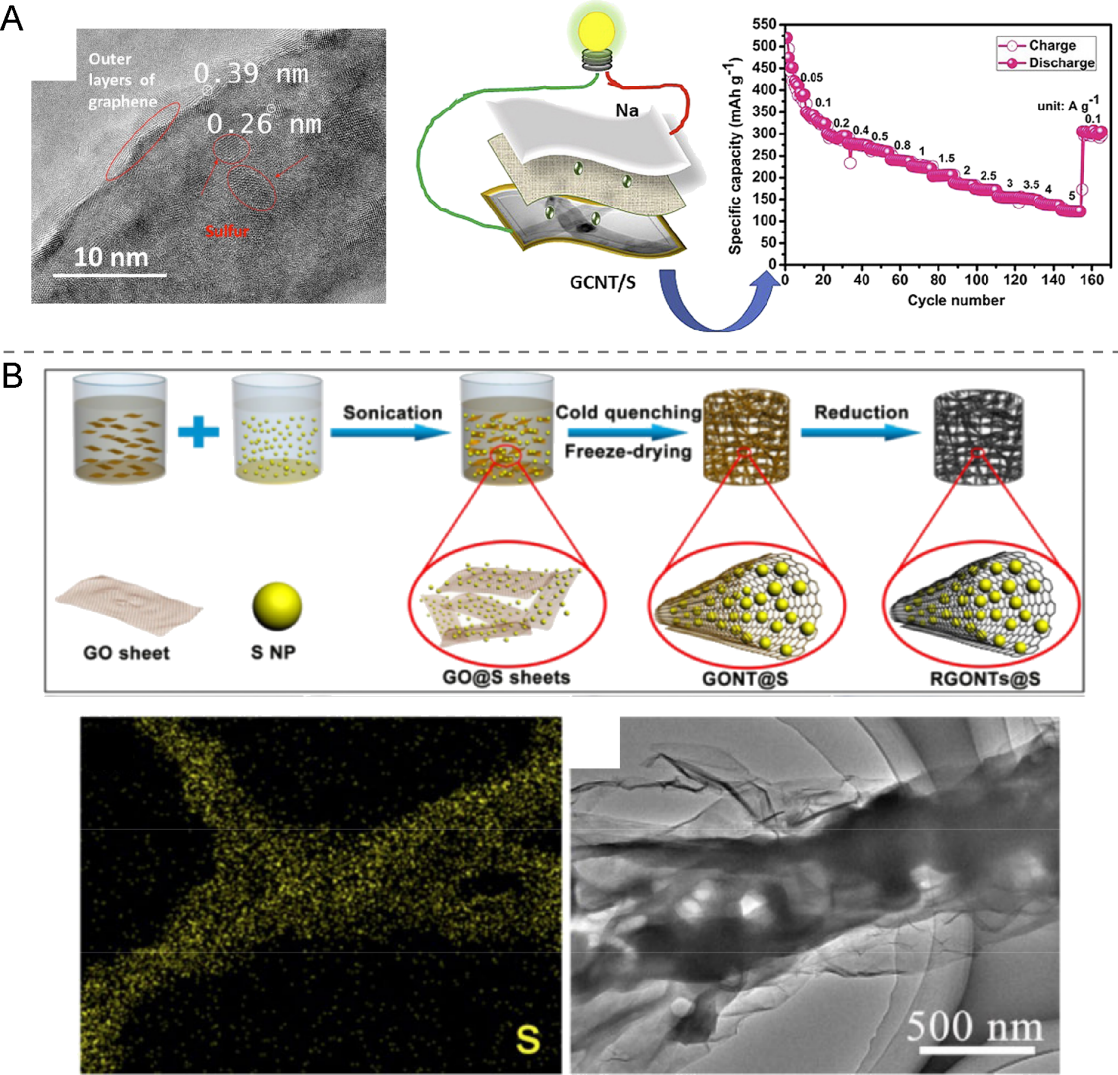
(A) HRTEM image of sulfur nanoparticles and graphene in GCNT/S and the cycle performance at 1 A·g^−1^. Figure 7A was reprinted from [54], Journal of Alloys and Compounds, vol. 818, by A. P. V. K. Saroja, M. Kamaraj, S. Ramaprabhu, "Strongly coupled sulfur nanoparticles on graphene-carbon nanotube hybrid electrode for multifunctional sodium and aluminium ion storage", article no. 152864. Copyright (2020), with permission from Elsevier. This content is not subject to CC BY 4.0. (B) Schematic illustration of sulfur NPs assembled with reduced graphene oxide nanotubes and flexible films prepared of these (RGONTs@S). SEM/EDX elemental S map and TEM image of RGONTs@S. Figure 7B is from [58] and was reprinted by permission from Springer Nature from the journal Nano Research ("Sulfur nanoparticles encapsulated in reduced graphene oxide nanotubes for flexible lithium-sulfur batteries" by K. Chen, J. Cao, Q. Lu, Q. Wang, M. Yao, M. Han, Z. Niu, J. Chen). Copyright 2018 Springer Nature. This content is not subject to CC BY 4.0.

There are different methods to incorporate sulfur nanoparticles and nanostructures in cathodes such as physical and chemical impregnation, mechanical mixing and self-assembly. Straightforward approaches are sulfur melt and vapor impregnation of suitable matrices. These routes require heating to temperatures above the melting (115 °C) and boiling (446 °C) point of sulfur, respectively. While the former methodology is more prone to generate rather thick and bulky sulfur layers, the latter can produce fine particles down to molecular sulfur species (S_8_) deposited on and within porous host materials [53]. Many cathodes of Na–S batteries are fabricated by the impregnation approach. In a recent work, 10 nm S NPs were synthesized inside carbon nanotubes by the melt diffusion method (Figure 7A) [54]. The resulting capacity of the Na–S battery after 2500 cycles at 1 A·g^−1^ was 80 mAh·g^−1^. Likewise, sulfur impregnation of hollow carbon nanocages rendered cathodes of 395 mAh·g^−1^ at 1 A·g^−1^ for 850 cycles [45]. However, important issues are the need for elevated temperatures, working under sealed Ar or N_2_ conditions, and the possibly inhomogeneous sulfur distribution within the host material. Therefore, also ball milling is being employed to produce fine sulfur particles (tens of nanometers) from sulfur powder in the presence of graphene [55]. One advantage of this method over vapor impregnation routes is that it can yield very high sulfur loads of up to 90 wt % resulting in a high cathode capacity of 555 mAh·g^−1^ at 5C [55].

Wet chemistry synthesis routes can also produce nanostructured sulfur. Lu et al. reported on the wet impregnation of carbon fiber cloth with sulfur dissolved in CS_2_ [56]. The process leads to sulfur deposition within the hollow lumen of the carbon fibers as well as on the external surface as thin film. A battery assembled with a metal Na anode had a capacity of 120 mAh·g^−1^ after 300 cycles at 167 mA·g^−1^ current density. While CS_2_ is the most efficient solvent for sulfur, it is also an extremely reactive and hazardous solvent.

Therefore, more benign solution methods for the synthesis of S NPs and sols are sought. In fact, such methods have been known for more than a hundred years now and can be distinguished in aqueous and organic solvent routes [57]. The so-called Weimarn sols are produced by dissolution of sulfur powder in ethanol or acetone-based solutions and the subsequent precipitation of sub-micrometer-size particles in water [57]. The so-called Raffo sols are obtained by a disproportionation reaction of Na_2_S_2_O_3_ in presence of concentrated H_2_SO_4_ [57]. Raffo sols are stable, aqueous colloidal dispersions of SO_3_^−^-capped sulfur NPs in the 100–500 nm range and could be readily assembled with other compounds such as cathode materials. Yet, this fabrication route of sulfur cathodes appears to be highly underinvestigated despite the advantages an aqueous solution-based synthesis route can offer. Instead, commercial S NPs are increasingly employed in cathodes, for instance, by Chen et al. [58], who wrapped reduced graphene oxide sheets around such NPs (Figure 7B). In this case, the cathode was assembled in a Li battery that delivered 490 mAh·g^−1^ after 500 cycles at 1C.

It can be noticed that the concept of discrete S NPs is much more extensively researched in Li–S batteries than in Na–S batteries [59–61], which can be possibly explained by the longer history of Li battery research. Qu et al. [60] precipitated 15 nm sized S NPs on V_2_O_5_ by acid hydrolysis of Na_2_S_2_O_3_. After encapsulation with graphene sheets the cathode had a discharge capacity of 215 mAh·g^−1^ at 2C after 2000 cycles. In another chemical method, S NPs were precipitated on rGO from a deep eutectic solvent consisting of choline chloride and Na_2_S_2_O_3_ [61]. This cathode material retained 900 mAh·g^−1^ over 100 cycles. A modified precipitation method for S NPs is flash nanoprecipitation using a confined impingement jet mixer, in which Na_2_S_2_O_3_, H_2_O, HCl, and a stabilizing copolymer (polyvinylpyrrolidone, PVP) form sub-micrometer-sized sulfur NPs within milliseconds [62]. The resulting sulfur–PVP composite cathode had a capacity of 808 mAh·g^−1^ after 50 cycles at 0.1C.

### Sodium metal-free anodes

**Figure 8:**
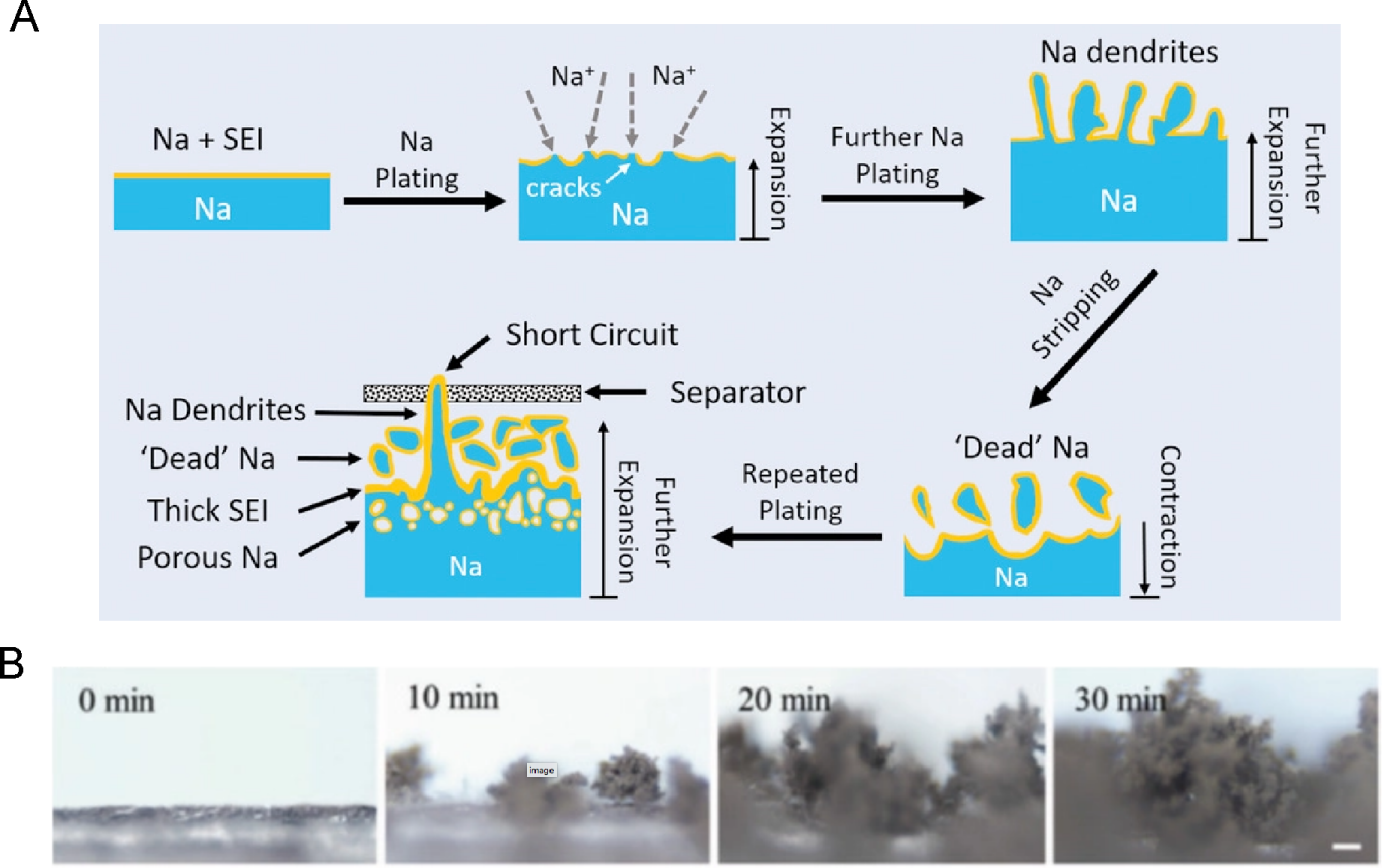
(A) Illustration of the Na dendrite formation mechanism during charging cycles, where Na is redeposited from Na^+^ on the anode (Na plating). The solid electrolyte interphase (SEI) formed on the Na anode is not capable of preventing the catastrophic dendrite growth. Figure 8A was reproduced from [10] (© 2019 B. Sun et al., published by WILEY‐VCH Verlag GmbH & Co. KGaA, Weinheim, distributed under the terms of the Creative Commons Attribution 4.0 International License, https://creativecommons.org/licenses/by/4.0). (B) Visualization of sodium dendrite growth in a carbonate electrolyte. Figure 8B was reprinted with permission from [65], X. Yang et al. "Anodes and Sodium-Free Cathodes in Sodium-Ion Batteries", Adv. Energy Mater., with permission from John Wiley and Sons Copyright © 2020 WILEY-VCH Verlag GmbH & Co. KGaA, Weinheim. This content is not subject to CC BY 4.0.

The reason for the widespread use of metal Na anodes in Na–S batteries is the very high capacity of 1165 mAh·g^−1^ of metallic Na and the low reduction potential of −2.71 V vs SHE. However, metallic Na is plagued by a range of severe drawbacks [63]. First of all, it is a very reactive element, which requires safe handling in inert atmosphere and storage in water-free petroleum. It can even react with aprotic solvents of electrolytes and compromise the performance and safety of the battery [64]. Other reasons for avoiding metal Na anodes are the pronounced dendrite formation and significant volume expansion and contraction during operation leading to performance loss [19,65]. Figure 8 illustrates the Na dendrite formation in the course of Na plating and stripping as the battery charges and discharges, respectively.

There are numerous strategies to mitigate the dendrite and reactivity issues of metal Na anodes. Amongst the most investigated approaches concerning the former are the controlled formation of protective solid electrolyte interphases (SEI) [66], while the latter is addressed by engineering of liquid and solid electrolytes [63]. Results show that these strategies have an undeniable positive influence on cycle stability and performance safety of sodium batteries [10]. Yet, there are currently also other strategies emerging that advocate metal Na-free anodes. These can be divided in depositing nanometric Na on a porous host material and inserting Na ions in a suitable host material. Host materials for the deposition of Na are porous scaffolds based on carbon and metal. The insertion of Na ions can be performed with certain metals, semimetals, phosphorous, and carbon allotropes.

#### Dispersed Na anodes

**Figure 9:**
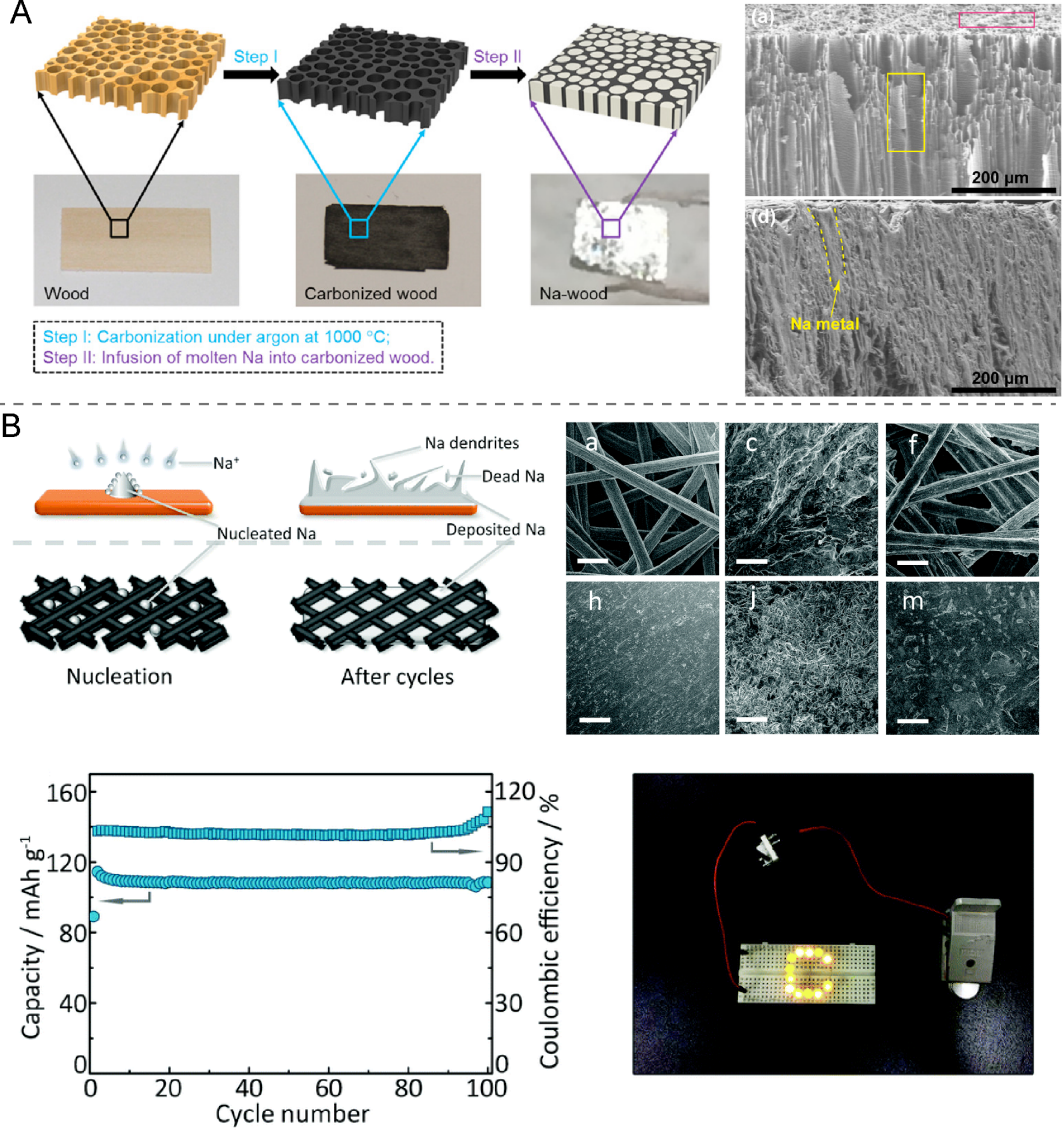
(A) Encapsulation of molten metallic Na into porous carbonized wood by a spontaneous infusion. SEM images of carbonized wood before and after melt infusion of metallic Na (yellow dotted line). Figure 9A was reprinted with permission from [67]. Copyright 2017, The American Chemical Society. (B) Schematic illustration of structural changes with Na depositing on Cu and CFP current collector during Na nucleation and growth. SEM images of Cu/CFP (upper row) and Cu (bottom row) after 30 plating/stripping cycles. The capacity and Coulombic efficiency of FeHCF with the CFP@Na anode. Figure 9B was reproduced from [69] with permission from the Chinese Chemical Society (CCS), Peking University (PKU), and the Royal Society of Chemistry. This content is not subject to CC BY 4.0.

In strict terms this approach is not Na metal-free as it relies on finely dispersed metallic Na. But the small dimensions of the Na phase result in battery behavior distinct from macroscopic metal Na foil anodes. There are two fabrication methods of dispersed Na anodes. In one liquid Na is soaked into a porous scaffold, while in the other Na ions plate the porous host material. An example of the former method is the use of carbonized wood, into the open pore system of which liquid Na is soaked (Figure 9A) [67]. Also, nanocarbon materials such as graphene and carbon aerogels, carbon microspheres, and mats, felts and papers based on carbon nanotubes and carbon fibers can also be efficiently soaked with Na and additionally provide bending and rolling flexibility, making them very attractive host materials [19,67–70].

Na can also be deposited in even finer structures by an electrochemical plating process. Herein, Na ions originating either from a metal Na electrode or from Na_2_S/composite cathodes [27] are deposited on an electro-conductive host material, where they nucleate and grow into extended but nanometric Na coatings. For instance, Zhang et al. [69] used carbon fiber paper (CFP) to grow a Na layer from a metal Na electrode (Figure 9B). In the case of carbon hosts it needs to be kept in mind that Na^+^ can also insert into the material as will be discussed further below. Therefore, the specific capacity of these electrodes depends on two mechanisms, the deposition/stripping of Na and the insertion/extraction of Na^+^. Metal scaffolds and meshes fabricated from Cu, Ni, Ni@Cu, or Al are other materials onto which Na can be plated. As shown in a recent work, porous Ni structures formed on Cu foil served as 3D current collector for plating Na [71]. Porous Al current collectors are also interesting Na plating substrates due to the lower weight and cost of Al compared to Cu and Ni. The resultant Al/Na anodes displayed high cycle stability (1000 cycles) with minimal Coulombic efficiency loss [72].

What is common to all these approaches is that finely dispersed Na, even in its metallic Na form, suppresses dendrite formation to a significant extent. The generally reported reason for this observation is the more even distribution of Na nuclei, the greatly reduced concentration polarization, and a more robust SEI [19]. Further manifestations of the advantage of nanometric Na is the avoidance of a mechanic breakdown of the anode as the volume expansion and pore formation during plating/stripping is minimized in Na nanostructures. Eventual stresses are also significantly better distributed and accommodated by the matrices, especially in the case of flexible ones.

#### Na alloys and intermetallics

Sodium is capable of forming alloys and intermetallic compounds with a range of elements at room temperature, most notably with Sb, Sn, P, Si, Ge, and Bi [73]. This way, considerable amounts of Na can be stored safely in intermetallic anodes, from which Na ions are reversibly released during discharging and charging processes. Among the most common alloying elements in SiBs are tin [74], antimony [75], and to a lesser degree phosphorous [76]. These elements can be either directly sodiated to form Na alloys or intermetallic compounds (M-Sn/Sb/P with M = Sn, Sb, P, Si, Bi, Cu, Ni, Fe, Zn).

**Figure 10:**
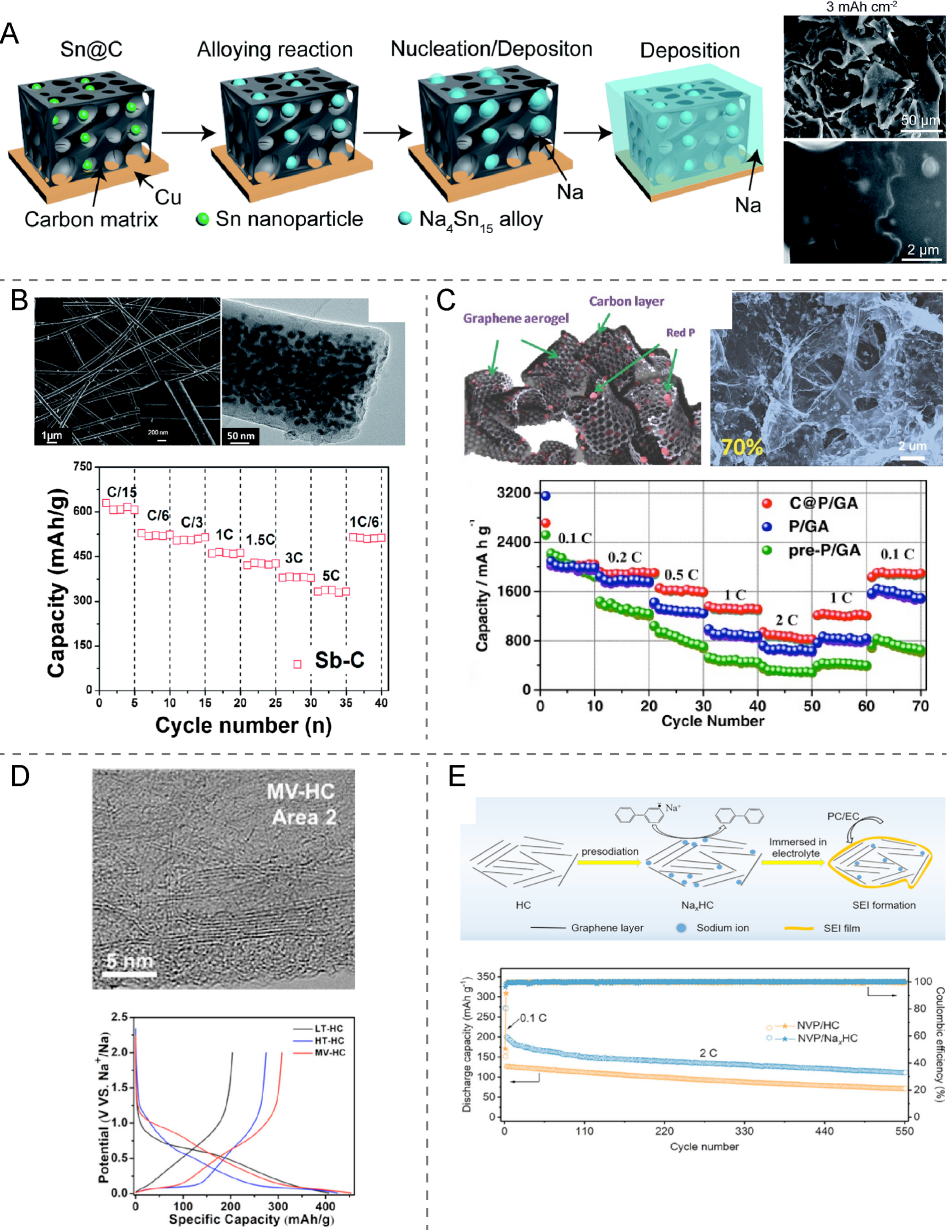
(A) Illustration of the sodiation process of a flexible Sn@C composite substrate and corresponding SEM images of the surface morphology upon Na plating with a current density of 2 mA·cm^−2^ and a capacity of 3 mAh·cm^−2^. Figure 10A was republished with permission of The Royal Society of Chemistry, from [77] ("Tin nanoparticles embedded in a carbon buffer layer as preferential nucleation sites for stable sodium metal anodes" H. Wang et. al., J. Mater. Chem. A, vol. 7, © 2019); permission conveyed through Copyright Clearance Center, Inc. This content is not subject to CC BY 4.0. (B) SEM and TEM images of the Sb–C nanofibers and the C-rate capability at various current rates. Figure 10B was republished with permission of The Royal Society of Chemistry, from [78] ("Sb–C nanofibers with long cycle life as an anode material for high-performance sodium-ion batteries" L. Wu et. al., Energy Environ. Sci., vol. 7, © 2013); permission conveyed through Copyright Clearance Center, Inc. This content is not subject to CC BY 4.0. (C) Schematic illustration of the 3D porous graphene/red P composite (C@P/GA), a SEM image of the composite with 70% of red P and the rate performance of the pre-P/GA, P/GA, and C@P/GA composites. Figure 10C was reproduced with permission from [81], H. Gao et al. "Integrated Carbon/Red Phosphorus/Graphene Aerogel 3D Architecture via Advanced Vapor-Redistribution for High-Energy Sodium-Ion Batteries", Adv. Energy Mater., with permission from John Wiley and Sons Copyright © 2016 WILEY-VCH Verlag GmbH & Co. KGaA, Weinheim. This content is not subject to CC BY 4.0. (D) TEM image of MV-HC and first-cycle galvanostatic sodiation/desodiation potential profiles of three hard carbons at a current rate of 20 mA/g. Figure 10D was reprinted with permission from [82]. Copyright 2018, The American Chemical Society. This content is not subject to CC BY 4.0. (E) Schematic illustration of pre-sodiated hard carbon (HC) including a SEI layer and the long-term cycling performance of the full cell at a constant rate of 2C. Figure 10E was reprinted with permission from [86]. Copyright 2020, The American Chemical Society. This content is not subject to CC BY 4.0.

Fully sodiated Sb, Sn, and P form the phases Na_3_Sb, Na_15_Sn_4_, and Na_3_P, which offer theoretical capacities of 660, 847 and 2596 mAh·g^−1^, respectively [73]. However, the measured values are usually somewhat lower due to cycle instability, slow sodiation kinetics, and SEI breakaway. The low cycle stability is caused by the considerable volume expansion in the range of 300–500% during sodiation of these elements, which threatens the integrity of the anode [73–74,76]. One solution to this drawback is down-sizing the active alloy to the nanometer scale in form of nanoparticles, nanorods, nanofibers, nanoarrays, and nanosheets, which are more tolerant to dilative stress [75–76]. In addition, these nanostructures can be incorporated into a flexible carbon matrix for further accommodation of mechanical strain (Figure 10A) [74,77]. The latter can be achieved, for instance, by electro-spinning of carbon–Sb nanofibers, which delivered up to 630 mAh·g^−1^ at a rate of C/15 [78] (Figure 10B). Further examples are alloy/carbon nanocomposites consisting of Sn/graphene [79], Sb/graphene [80], Sn/carbon foams [79], red P/carbon aerogel [81] (Figure 10C), and Sn/carbon spheres [79] with a capacity retention of 70–90% over 100–500 cycles [73,75–76].

Sodiation of intermetallic compounds (M-Sn/Sb/P) is an interesting alternative as these intermetallic phases often show higher cycle stability than the pure elements and the respective alloys. This can be attributed to more effective mitigation mechanisms for the volume expansion [74]. Some intermetallic compounds also show higher capacities. For instance, a Sb anode with 7% of Si can reach a maximum capacity value as high as 663 mAh·g^−1^ after 140 cycles in a SiB, while the pure Sb anode delivered 625 mAh·g^−1^ [26]. Also, silicon itself is also another promising material for sodium alloy anodes as its theoretical Na storage capacity of 954 mAh·g^−1^ (NaSi phase) even exceeds the one of tin (847 mAh·g^−1^) [26]. The low electrochemical reactivity and structural stability of crystalline bulk silicon during sodiation can be overcome by hybridation of nanosized Si with carbon fibers, after which Zhang et al. measured 200 mAh·g^−1^ after 2000 cycles [83].

The investigation into phosphorous as anode material for SiBs is motivated by the highest capacity value of P (2596 mAh·g^−1^), which is even higher than that of metal Na [9]. However, also phosphorous anodes suffer from large volume expansion (up to 490%), but in addition also from low electrical conductivity [76]. The use of amorphous (red) phosphorous can lessen the expansion problem and extend cycle life. For instance, a red phosphorous/graphene anode delivered 1095 mAh·g^−1^ after 200 cycles at 1C [81]. It has also triggered research in a wider range of 2D materials as suitable and low-expansion anodes, starting with layered black phosphorous [73,76]. From there, exfoliated sheets of 2D allotropes came to scrutiny, such as antimonene, silicene, and phosphorene [73,83–84]. These sheets are usually integrated with graphene and other conducting carbon nanomaterials to afford mechanical support, flexibility, and electrical conductivity, which results in high capacity values (500–2000 mAh·g^−1^) over at least 100 cycles [73,75–76,83].

The majority of the alloy anodes discussed in this section were tested and employed in sodium-ion batteries with diverse types of oxide and phosphate cathodes. However, they are now also increasingly incorporated in sodium-ion–sulfur batteries. Lee et al. were amongst the first to proof this concept by employing a sodiated Sn–C anode and hollow C spheres infused with elemental sulfur as cathode [20]. The full cell rendered 550 mAh·g^−1^ at 167 mA·g^−1^ within a 0.1–1.8 V voltage limit, albeit after 12 cycles the capacity dropped to 450 mAh·g^−1^. Hence, there is much room for further investigation and improvement.

#### Hard carbon anodes

A somewhat more conventional approach to Na metal-free anodes is the use of hard carbon, also termed non-graphitizable carbon [82,85]. These are disorganized carbon materials with turbostratic nanoscale domains produced by pyrolysis of biomass, also including carbon black and other amorphous carbons. While Na^+^ does not insert in graphite in contrast to Li^+^, hard carbon can store considerable amounts of sodium in the range of 300 mAh·g^−1^ (Figure 10D) [82]. For their use in sodium batteries hard carbon materials can be pre-sodiated prior to the cell assembly. In a recent work, Liu et al. chemically pre-sodiated hard carbon using sodium biphenyl [86]. The SiB full cell showed a discharge capacity of 100 mAh·g^−1^ at 2C after 550 cycles (Figure 10E). Hard carbons can also be sodiated during the first charging cycle. Bloi et al. synthesized a porous Na_2_S/carbon cathode, which they coupled with a hard carbon electrode [27]. During the initial charging, Na^+^ plated the hard carbon, which rendered a fully sodiated anode. The Na–S full cell delivered 350 mAh·g_S_^−1^ in the first cycle and 130 mAh·g_S_^−1^ after ten cycles. This represents another example of successfully applying Na metal-free anode strategies for RT Na–S batteries.

### Relevant recent patents on RT Na–S batteries

Patents are a useful indicator for the innovation capacity of a scientific area and the potential commercialization of a technology. Therefore, this review includes a patent review on RT Na–S batteries to assess the maturity of these devices and elucidate possible technology bottlenecks. An overview search in ESPACENET [87], the European Patent Office platform, with the term “sodium-battery” showed 1451 results, with a prominent increase in the number of documents produced in the last 10 years. It is interesting to note that the major applicant was Toyota Motor Company, alongside with other relevant companies, such as Nanotek Instruments, Samsung Electronics and General Electric. This emphasizes the relevance of applications and commercial use of the research in this area. The search results were reduced to half (703) and to a quarter (358) when “room-temperature” and additionally “sulfur or sulphur” were added to the search query, respectively, leaving the abovementioned companies still as the main applicants. The additional inclusion of the term “nanoparticle or nanoparticles” significantly reduced the number of documents to just 56. It is noteworthy that, in this case, the documents are all dated from the last decade and also that Nanotek Instrument is the only of the previous companies that remains involved, appearing as the most relevant applicant with 17 documents.

A closer revision of the patents reveals that just about 50 of them could be properly considered relevant to Na–S batteries, though. In certain cases, the protected technology may also be applied to other types of batteries, typically lithium batteries. It is worth mentioning that most of the patents are in connection with companies, revealing the high importance of the topic from a technological and applicative point of view. The aspects covered by these patents ranged from the protection of complete devices to just covering specific materials and/or some components, mainly in relation to the electrodes. Table 2 collects information of some of the patents focused on the protection of devices. Most of these patents have been applied by Nanotek Instruments and include diverse designs of devices and processes for fabrication. The patents are very large in protecting the use of materials and components that may include a large variety of nanoparticles and nanomaterials, often related to the use of graphene and other nanoscale carbon materials as components of the electrode materials. A few patents are also from Broadbit Batteries OY. In this case, applications related to electrical vehicles were the main focus.

**Table 2:** Selection of patents protecting devices related to RT Na–S batteries and including nanotechnology aspects.

Patent publication number/date (ref.)	Title	Priorities/ Applicant	Area	Nanotechnology-related content

WO2017048341A1/ 2017-03-23 [88]	Alkali metal or alkali-ion batteries having high volumetric and gravimetric energy densities	2015-09-14/ B. Z. Jang, Nanotek Instruments Inc. & A. Zhamu	Alkali metal-ion battery, comprising an anode and cathode having the anode and cathode active material dispersed in a liquid electrolyte disposed in pores of a 3D porous anode/cathode current collector; and a separator disposed between the anode and the cathode	Components of the electrodes may include nanoparticles and diverse types of nanostructured materials (nanowires, nanodiscs, nanoribbons, nanoplatelets, nanocoatings, or nanosheets), for instance, graphene
WO2017055678A1/ 2017-04-06 [89]	Electrochemical secondary cells for high-energy or high-power battery use	2015-09-30/ Broadbit Batteries OY	Electrochemical cell for a secondary battery, preferably for use in an electric vehicle	The electrochemical cell includes a cathode and an anode and an electrolyte that includes one or more nitrogen-containing sol precursors and a salt comprising sodium, and in which the cathode material may contain diverse carbon nanoparticles
WO2017123544A1/ 2017-07-20 [90]	Alkali metal-sulfur batteries having high volumetric and gravimetric energy densities	2016-01-15/ B. Z. Jang, Nanotek Instruments Inc., Z. Aruna	Design of alkali metal (Li or Na)-sulfur battery, wherein the active cathode material is dispersed in an electrolyte and a conductive porous structure acting as a 3D scaffold	Description of the battery where the active cathode material is sulfur, polysulfide, sulfur–polymer composite, sulfur–carbon composite, sulfur-graphene composite, or a combination thereof
WO2017123546A1/ 2017-07-20 [91]	Method of producing alkali metal or alkali-ion batteries having high volumetric and gravimetric energy densities	2016-01-15/ B. Z. Jang, Nanotek Instruments Inc. & A. Zhamu	Process for producing an alkali metal battery, comprising multiple conductive porous layers, multiple wet anode layers of an active anode material mixed with a liquid electrolyte, and multiple wet cathode layers of an active cathode material mixed with a liquid electrolyte; stacking and consolidating a desired number of the porous layers and a desired number of wet anode/cathode layers to form an anode/cathode electrode; placing a porous separator layer in contact with the electrode and assembling all the components to produce the battery	Components of the electrodes may include nanoparticles and diverse types of nanostructured materials (nanowires, nanodiscs, nanoribbons, nanoplatelets, nanocoatings, or nanosheets), for instance, graphene
WO2017149204A2/ 2017-09-08 [92]	Rechargeable sodium cells for high energy density battery use	2016-03-04/ Broadbit Batteries OY	Electrochemical cell for an energy-dense rechargeable battery, including a solid metallic sodium anode	Description of a sodium rechargeable high energy density battery that includes the use of SO_2_ in the electrolyte
WO2018118800A1/ 2018-06-28 [93]	Flexible and shape-conformal cable-type alkali metal batteries	2016-12-20/ Nanotek Instruments Inc.	A cable-shaped alkali metal battery consisting of an electrode (a porous rod), a porous separator wrapped around, a second electrode wrapped around or encasing the porous separator, and a protective casing or packing tube wrapping	Components of the electrodes may include nanoparticles and diverse type of nanostructured materials (nanowires, nanodiscs, nanoribbons, nanoplatelets, nanocoatings, or nanosheets), for instance, hollow carbon nanowires or graphene
WO2018208660A1/ 2018-11-15 [94]	Rolled alkali metal batteries and production process	2017-05-08 & 2017-11-20/ Nanotek Instruments Inc.	Protect a rolled alkali metal (Li, Na, K) battery that comprises an anode, a cathode, an alkali metal ion-conducting separator, and an alkali metal ion-containing electrolyte in ionic contact with the anode and the cathode, wherein the anode and cathode contain a wound roll of an electrode active material substantially perpendicular to the separator plane	The composition of the electrode materials may include diverse types of nanoparticles including graphene
WO2018217274A1/ 2018-11-29 [95]	Alkali metal battery having a deformable quasi-solid electrode material	2017-05-24/ Nanotek Instruments Inc.	Alkali metal cell having a quasi-solid electrode, by combining a quantity of an active material, a quantity of an electrolyte, and a conductive additive to form a deformable and electrically conductive electrode material containing conductive filaments, forms a 3D network of electron-conducting pathways that can be deformed into an electrode shape without interrupting the 3D network pathways	The conductive filaments are selected from carbon nanofibers, graphite nanofibers, carbon nanotubes, metal nanowires, amongst other nanoparticles. Additionally, other elements of the cell may also contain diverse types of nanoparticles or nanostructured materials
WO2018222348A1/ 2018-12-06 [96]	Shape-conformable alkali metal battery having a conductive and deformable quasi-solid polymer electrode	2017-05-30 & 2017-05-31/ Nanotek Instruments Inc.	Method of preparing an alkali metal cell, comprising a deformable and conductive electrode material, containing conductive filaments included into a quasi-solid polymer electrode and a second electrode	Components in the electrodes may include nanoparticles and diverse types of nanostructured materials (nanowires, nanodiscs, nanoribbons or nanoplatelets, nanocoatings, or nanosheets), for instance, hollow carbon nanotubes
WO2018222349A1/ 2018-12-06 [97]	Shape-conformable alkali metal-sulfur battery	2017-06-02/ Nanotek Instruments Inc.	Design of alkali metal-sulfur cell including a quasi-solid cathode of a sulfur-containing material, alkali salt electrolyte conductive filaments to produce a 3D network of electron-conducting pathways	The composition of the elements of the battery includes the use of diverse nanomaterials and nanostructured materials, for instance, the use of conductive filaments such as carbon nanotubes, carbon nanofibers, nanostructured or porous disordered carbon materials
WO2019005299A1/ 2019-01-03 [98]	Shape-conformable alkali metal-sulfur battery having a deformable and conductive quasi-solid electrode	2017-06-30/ Nanotek Instruments Inc.	Design of alkali metal-sulfur cell including a quasi-solid cathode of a sulfur-containing material, alkali salt electrolyte conductive filaments to produce a 3D network of electron-conducting pathways	Use of conductive filaments such as carbon nanofibers, carbon nanotubes, metal nanowires, graphene, and others
WO2019045907A1/ 2019-03-07 [99]	Continuous process for producing electrochemical cells	2017-08-28/ Nanotek Instruments Inc.	Process for producing an electrochemical cell, comprising a continuously depositing a wet cathode or anode active material mixture onto a cathode or anode current collector, and then combining the cathode electrode and the anode electrode to form the cell	The anode active material contains an alkali intercalation compound selected from hollow carbon nanowires, amongst other carbonaceous materials

A large part of the patents protects certain elements of the battery electrodes (Table 3). Some of the patents protect the production of specific materials for application as electrode, including the production and/or use of different types of nanoparticles, such as oxides and carbonaceous materials that can be incorporated into the anode and/or cathode material to increase the efficiency. In other cases, patents also include aspects dealing with the construction of electrodes, in which the design of precise structures, for instance, 3D networks, favors the presence of specific conducting pathways that improve the performance of the electrode. Again, a large number of these patents were applied by companies from different industry fields.

**Table 3:** Selection of representative patents dealing with RT Na–S batteries and nanoparticles and nanotechnology, which protect aspects related to the electrodes.

Patent publication number (ref.)	Title	Priority/ Applicant	Area	Nanotechnology-related content

WO2012128262A1/ 2012-09-27 [100]	Sodium secondary cell electrode and sodium secondary cell	2011-03-24 & 2011-10-12/ T. Ishikawa; S. Komaba; S. Kuze; Y. Matsuura; W. Murata; Sumitomo Chemical Co.; Univ. Tokyo Science Education Found & N. Yabuuchi	Sodium secondary cell electrode contains tin powder as an electrode active material and other forming agents of polymeric type	Sn particles can be of nanometric size
WO2012151094A2/ 2012-11-08 [101]	Composite materials for battery applications	2011-05-04/ A. Abouimrane, K. Amine, J. Ren, UChicago Argonne LLC, J. Yang	A process for producing nanocomposite materials for use in batteries includes electroactive materials are incorporated within a nanosheet host material	A gaseous electroactive material precursor interacts with a carbonaceous, exfoliated nanosheet material to form a nanocomposite material, including the use of graphene as exfoliated nanosheet carbonaceous material
WO2014083135A1/ 2014-06-05 [102]	Tin-based anode material for a rechargeable battery and preparation method	2012-11-30/ Belenos Clean Power Holding AG	Tin-based nanoparticles as anode of sodium and lithium batteries	Development of tin mixed oxides nanoparticles for using as anode in sodium batteries
US10320000B2/ 2019-06-11 [103]	Pyrolytic carbon black composite and method of making the same	2013-07-18 & 2016-02-29/ UT-Battelle LLC	Method for preparing sulfonated-carbon material for using in electrodes of lithium-ion or sodium-ion battery	The carbon source to produce the electrode material includes carbon reinforcing agents that may consist of carbon nanoparticles
US2017155140A1/ 2017-06-01 [104]	Antimony-based anode material for rechargeable batteries and preparation method	2013-11-28/ Belenos Clean Power Holding AG	Antimony-based nanoparticles as anode of lithium and sodium batteries	Development of antimony mixed oxides nanoparticles for using as anode in sodium batteries
WO2017062197A1/ 2017-04-13 [105]	Continuous process for producing electrodes and alkali metal batteries having ultra-high energy densities	2015-10-08/ B. Z. Jang, Nanotek Instruments Inc. & A. Zhamu	Method for producing A process for continuously producing an electrode for an alkali metal battery, where the electrode material may include diverse type of nanoparticles such as graphene nanoplatelets	The method includes various steps: continuously feeding an electrically conductive porous layer to an anode or cathode material impregnation zone, impregnating a wet anode or cathode active material mixture to form an anode or cathode electrode, and supplying a protective film to cover the electrode
WO2017172044A2/ 2017-10-05 [103]	Pyrolytic carbon black composite and method of making the same	2016-02-29/ UT-Battelle LLC	Method for preparing sulfonated-carbon material for using in electrodes of lithium-ion or sodium-ion battery	The carbon source to produce the electrode material includes carbon reinforcing agents that may consist of carbon nanoparticles
US2019270678A1/ 2019-09-05 [106]	New process for producing highly carbonaceous materials and the highly carbonaceous material obtained	2016-10-28 & 2017-10-26/ Arkema France	Process for the production of highly carbonaceous material including steps of carbonization of fibers covered with a cyclic organic or aromatic compound to produce a highly carbonaceous material of possible application in alkali batteries	The precursors and reagents used to produce the highly carbonaceous material include nanocellulose and diverse carbonaceous nanofillers
WO2019108343A1/ 2019-06-06 [107]	Anode particulates or cathode particulates and alkali metal batteries containing same	2017-11-30 & 2017-12-05/ Nanotek Instruments Inc.	Electrodes, anode and cathode based on the combination of components forming a three dimensional network of electron-conducting pathways in contact with the electrode active material	The electrode components may include diverse types of nanoparticles, nanowires, nanofibers, nanotubes, nanosheets, nanobelts, nanoribbons, nanodiscs, nanoplatelets, or nanohorns, for instance, carbon nanofibers or carbon nanotubes, hollow carbon nanowires, nanospheres, or graphene
US10637043B2/ 2020-04-28 [108]	Anode particulates or cathode particulates and alkali metal batteries containing same	2017-11-30/ Nanotek Instruments Inc. & Global Graphene Group Inc.	Electrode material based on the combination of components forming a three dimensional network of electron-conducting pathways	The electrode includes an active material capable of reversibly absorbing sodium ions, an electron-conducting material, and a sodium ion-conducting electrolyte
US10873083B2/ 2020-12-22 [109]	Anode particulates or cathode particulates and alkali metal batteries	2017-11-30 & 2018-01-02/ Global Graphene Group Inc.	Anode and cathode including nanoparticles for an alkali metal battery where the particulate can be of any shape, but preferably spherical or ellipsoidal in shape	The electrode material contains nanoparticles, nanowires, nanofibers, nanotubes, nanosheets, nanobelts, nanoribbons, nanodiscs, nanoplatelets, or nanohorn-shaped particles
US2019173079A1/ 2019-06-06 [110]	Method of producing participate electrode materials for alkali metal batteries	2017-12-05 & 2018-01-02/ Nanotek Instruments Inc.	Method of producing anode or cathode particulates for an alkali metal battery	The method is characterized for converting a said slurry into multiple anode/cathode particulates having dimensions on the nano/microscale
US10797313B2/ 2020-10-06 [111]	Method of producing anode or cathode particulates for alkali metal batteries	2017-12-05/ Nanotek Instruments Inc. & Global Graphene Group Inc.	Method of producing anode or cathode particulates for an alkali metal battery including particles of the active material, the electron-conducting material forming a 3D network, and an electrolyte	The combination of particulate components implies the use of pan-coating, air-suspension coating, centrifugal extrusion, vibration nozzle, spray-drying, interfacial polycondensation or interfacial cross-linking, in situ polymerization, matrix polymerization methodos, or a combination thereof
WO2019135827A1/ 2019-07-11 [112]	Anode particulates or cathode particulates for alkali metal batteries	2018-01-02/ Nanotek Instruments Inc.	Electrode, anode and cathode, materials formed of particles of the electrode material, an electron-conducting material, and an alkali salt with an optional polymer or its monomer, but without a liquid solvent, forming a 3D network of electron-conducting pathways, for sodium and lithium battery applications	The particulate electrode material may contain components as nanoparticles, nanowires, nanofibers, nanotubes, nanosheets, nanobelts, nanoribbons, nanodiscs, nanoplatelets, or nanohorns having a thickness or diameter from 0.5 nm to 100 nm, and it could also include graphene as electron-conducting component
CN109437123A/ 2019-03-08 [113]	Selenium-doped ferrous disulfide carbon-coated composite material and preparation and application methods thereof	2018-10-16/ Zhongshan Gaorong New Energy Tech. Co. Ltd.	Preparation and application methods of selenium-doped ferrous disulfide carbon-coated composite material	Application of a selenium-doped ferrous disulfide carbon-coated composite as a negative electrode material in sodium-ion batteries

Finally, Table 4 includes several patents selected as representative examples of other protected items of interest related to Na–S batteries. For instance, the development of polymer electrolytes, additives, salts, or specific materials that can be used in the preparation of components and conformation of elements of the battery.

**Table 4:** Other patents of interest related to RT Na–S batteries.

Patent publication number (ref.)	Title	Priority/ Applicant	Area	Relevance for Na–S batteries

EP3422438A1/ 2019-01-02 [114]	Solid polymer electrolyte based on modified cellulose and its use in lithium or sodium secondary batteries	2017-06-28/ Fundación Centro de Investigación Cooperativa de Energías Alternativas CIC Energigune Fundazioa	Solid polymer electrolyte based on modified cellulose incorporating by covalent grafting the anion of an organic sodium salt or lithium salt	Alternative polymer electrolyte for application in batteries
WO2019010474A1/ 2019-01-10 [115]	Electrospinning of PVDF-HFP: novel composite polymer electrolytes (CPES) with enhanced ionic conductivities for lithium-sulfur batteries	2017-07-07/ Univ. Pittsburgh Commonwealth Sys. Higher Education	Polymer electrolyte separator that comprises electrospun nanofibers, a lithium/magnesium/sodium solid or liquid electrolyte and nanoparticle filler (metal oxides and metal non-oxide, groups III, IV, or V)	Composite polymer electrolyte separators for lithium batteries and extended to sodium and magnesium batteries
US2020251781A1/ 2020-08-06 [116]	Non-aqueous electrolytes for electrochemical cells	2019-02-04/ UChicago Argonne LLC	A non-aqueous electrolyte comprising a salt, a non-aqueous solvent, and a compound containing S(O)-or S(O)_2_- groups for diverse uses including Na–S-batteries	The electrolyte can be used in electrochemical devices that may include diverse types of nanomaterials in some of their components, for instance carbon nanotubes, carbon nanofibers, graphene, tin nanoparticles, and others
WO2020006642A1/ 2020-01-09 [117]	Glycidyl-containing polymers, polymer compositions comprising them and their use in electrochemical cells	2018-07-06/ Hydro Quebec & Murata Manufacturing Co.	Glycidyl-containing polymers and polymer compositions for uses in electrode materials and/or as coatings for battery components	The polymer may be used in the preparation of electrode materials to disperse nanoparticles
US2014205883A1/ 2014-07-24 [118]	Reactive separator for a metal-ion battery	2012-03-28 and others/ Sharp Lab. of America Inc.	A reactive separator for a metal-ion battery made up of a reactive layer that is chemically reactive to alkali or alkaline earth metals, and has a first side and a second side	The reactive layer may be formed as a porous membrane (carbon or a porous polymer) embedded with reactive components or is formed as a polymer gel embedded with reactive components
KR102001454B1/ 2019-07-18 [119]	The preparation method of multi-layer core–shell nano particles comprising porous carbon shell and core–shell nano particles thereby	2017-09-27/ Korea Institute of Energy Research	Methodology for preparation of core–shell nanoparticles of various components and compositions	Possible use of core–shell nanoparticles as components of electrode materials in lithium and sodium batteries
WO2013073259A1/ 2013-05-23 [120]	High-purity parastyrene sulfonic acid (salt); polystyrene sulfonic acid (salt) using same; dispersant, conductive polymer dopant, aqueous nanocarbon material dispersion and aqueous conductive polymer dispersion each using polystyrene sulfonic acid (salt); and method for producing polystyrene sulfonic acid (salt)	2011-11-16/ H. Matsunaga; S. Ozoe; Tosoh Organic Chemical Co. Ltd. & K. Yamanoi	Novel polystyrene sulfonic acid (salt) useful as a dispersant for producing an aqueous dispersion of a nanocarbon material or an aqueous dispersion of a conductive polymer	The material can be used as a dispersant for producing carbon nanomaterials, such as carbon nanotubes, graphene, or fullerenes that can be used as electrode protective film and separator for sodium secondary battery
CN111517374A/ 2020-08-11 [121]	Preparation method of Fe_7_S_8_/C composite material	2020-04-20/ Jixi Weida New Material Tech Co. Ltd.; Univ Science & Technology Liaoning	Method for preparation an iron sulfide/carbon composite material for potential use as electrode in lithium and potentially other alkaline-ion batteries	Combination of sulfide nanoparticles and the carbon component improve the conductivity of the anode materials and also act as a structural buffer
US2014023922A1/ 2014-01-23 [122]	Manufacturing method of an electrode for an electrochemical element	2011-01-21 & others/ Y. Isshiki Yasuhiro; Y. Wakizaka & Zeon Corp.	Method to produce an electrode for an electrochemical element having a superior adhesion	The electrode material (a mixed powder or composite particles) includes an alkaline metal powder for uses in diverse alkaline batteries

The analysis of the patent search results clearly shows a lack of specific patents dealing with the production of cathode materials involving sulfur nanoparticles or anodes consisting of nanostructured sodium, though from the ambiguous language of these documents it is challenging to ascertain if such issues were addressed. It must be noted that only patents registered by companies were analyzed in this study. It is possible that academic institutions may have already protected methodologies or materials introduced in this review. Besides, it could be expected that the increasing research and technological relevance will translate in an increasing number of patents and actual transfer to the productive sector.

## Conclusion

The revival of Na–S batteries has triggered enormous research within this field. Important drivers are, besides the high theoretical capacity and energy density values, aspects of sustainability, environmental impact, and geo-economic concerns. Sodium and sulfur are amongst the most abundant elements on Earth, widely available and with a relatively small ecological footprint. They also do not require the same amount of rare and socially contested metals as LiBs. The substitution of cobalt-based electrocatalytic NPs in RT Na–S with less critical transition metal and noble metal NPs would further improve their environmental performance. It is expected that sodium batteries will replace LiBs at some point in the future and sustain further electrification of the daily life, an important pillar of sustainable development in a post-carbon society. However, important obstacles have to be overcome to make this vision come true, especially in the case of RT Na–S batteries. While they share important electrochemical challenges with Li–S and sodium-ion batteries, some aspects are more pronounced or unique to RT Na–S. The polysulfide shuttle effect is a serious drawback lowering the life cycle stability and Coulombic efficiency. Likewise, the severe dendrite growth in Na–S is much more prominent than in Li–S batteries, affecting the safety of the batteries as well as reducing the cycling performance and capacity. More specific issues are the slow reaction kinetics between Na and S and the low electrical conductivity of sulfur and Na_2_S. Volume expansion that leads to material breakdown is a threat both at the cathode and the anode side, especially in case of sodiated alloys. It was shown that down-sizing the active components on both electrodes is a viable strategy to mitigate many of these issues. Nanostructured and nanoparticulated sulfur is easily entrapped in electroconducting matrices, which reduces the shuttle effect, increases the cathode conductivity, accommodates mechanical stress from volume expansion. Also, the high surface area accelerates reaction kinetics. A similar strategy can be pursued at the Na anode, where nanosized Na alloys can reduce dilative material failure and Na dendrite growth. Especially the focus on Na metal-free anodes is foreseen as an important element on the way to commercial RT Na–S batteries. Since much progress has been achieved in the understanding of sodium intercalation in anode materials of SiBs, this knowledge should now be transferred to RT Na–S batteries to increase their safety and applicability.

The patent survey also revealed quite clearly the gap that exists between academic and industrial RT Na–S battery research, where the former flourishes with a diversity of concepts and material designs, while the latter is much more narrow in terms of material development. For instance, sulfur nanoparticles or nanostructured sulfur compounds do not appear in Na–S patents, whereas they do in some Li–S patents. It is also striking that there are no commercial RT Na–S batteries as of today, which underscores the long way to go for this technology. While the challenges are still numerous and severe, a breakthrough would allow for more efficient energy storage for meeting the sustainable development goals.
